# Phytochemical analysis, *in-vitro* and *in-silico* study of antiproliferative activity of ethyl acetate fraction of *Launaea cornuta* (Hochst. ex Oliv. & Hiern) C. Jeffrey against human cervical cancer cell line

**DOI:** 10.3389/fphar.2024.1399885

**Published:** 2024-06-28

**Authors:** Inyani John Lino Lagu, Dorothy Wavinya Nyamai, Sospeter Ngoci Njeru

**Affiliations:** ^1^ Department of Molecular Biology and Biotechnology, Pan African University Institute for Basic Sciences, Technology and Innovation, Nairobi, Kenya; ^2^ Department of Biochemistry, School of Biomedical Sciences, College of Health Sciences, Jomo Kenyatta University of Agriculture and Technology, Nairobi, Kenya; ^3^ Centre for Traditional Medicine and Drug Research (CTMDR), Kenya Medical Institute (KEMRI), Nairobi, Kenya

**Keywords:** *Launaea cornuta*, cervical cancer, phytochemicals, antiproliferative, network analysis, molecular docking, cytotoxicity, anticancer activity

## Abstract

**Introduction:** Cervical cancer is one of the leading causes of death among women globally due to the limitation of current treatment methods and their associated adverse side effects. *Launaea cornuta* is used as traditional medicine for the treatment of a variety of diseases including cancer. However, there is no scientific validation on the antiproliferative activity of *L. cornuta* against cervical cancer.

**Objective:** This study aimed to evaluate the selective antiproliferative, cytotoxic and antimigratory effects of *L. cornuta* and to explore its therapeutical mechanisms in human cervical cancer cell lines (HeLa-229) through a network analysis approach.

**Materials and methods:** The cytotoxic effect of *L. cornuta* ethyl acetate fraction on the proliferation of cervical cancer cells was evaluated by 3-(4, 5-dimethylthiazol-2-yl)-2, 5-diphenyltetrazolium bromide (MTT) bioassay and the antimigratory effect was assessed by wound healing assays. Compounds were analysed using the qualitative colour method and gas chromatography-mass spectroscopy (GC-MS). Subsequently, bioinformatic analyses, including the protein-protein interaction (PPI) network analysis, Gene Ontology (GO), and Kyoto Encyclopaedia of Genes and Genomes (KEGG) analysis, were performed to screen for potential anticervical cancer therapeutic target genes of *L. cornuta.* Molecular docking (MD) was performed to predict and understand the molecular interactions of the ligands against cervical cancer. Reverse transcription-quantitative polymerase chain reaction (RT-qPCR) was performed to validate the network analysis results.

**Results:** *L. cornuta* ethyl acetate fraction exhibited a remarkable cytotoxic effect on HeLa-229 proliferation (IC_50_ of 20.56 ± 2.83 μg/mL) with a selectivity index (SI) of 2.36 with minimal cytotoxicity on non-cancerous cells (Vero-CCL 81 (IC_50_ of 48.83 ± 23.02). The preliminary screening revealed the presence of glycosides, phenols, saponins, terpenoids, quinones, and tannins. Thirteen compounds were also identified by GC-MS analysis. 124 potential target genes associated with the effect of *L. cornuta* ethyl acetate fraction on human cervical cancer were obtained, including AKT1, MDM2, CDK2, MCL1 and MTOR were identified among the top hub genes and PI3K/Akt1, Ras/MAPK, FoxO and EGFR signalling pathways were identified as the significantly enriched pathways. Molecular docking results showed that stigmasteryl methyl ether had a good binding affinity against CDK2, ATK1, BCL2, MDM2, and Casp9, with binding energy ranging from −7.0 to −12.6 kcal/mol. Tremulone showed a good binding affinity against TP53 and P21 with −7.0 and −8.0 kcal/mol, respectively. This suggests a stable molecular interaction of the ethyl acetate fraction of *L. cornuta* compounds with the selected target genes for cervical cancer. Furthermore, RT-qPCR analysis revealed that CDK2, MDM2 and BCL2 were downregulated, and Casp9 and P21 were upregulated in HeLa-229 cells treated with *L. cornuta* compared to the negative control (DMSO 0.2%).

**Conclusion:** The findings indicate that *L. cornuta* ethyl acetate fraction phytochemicals modulates various molecular targets and pathways to exhibit selective antiproliferative and cytotoxic effects against HeLa-229 cells. This study lays a foundation for further research to develop innovative clinical anticervical cancer agents.

## Introduction

Cervical cancer is a major global health disease that affects women (Drokow et al., 2022). It is one of the leading causes of cancer death among women and the fourth most common cancer worldwide (Revathidevi et al., 2021). Human papillomavirus is the primary aetiological driver in carcinogenesis (Balasubramaniam et al., 2019). Not all human papillomavirus (HPV) infections in women result in cervical cancer. High-risk HPV genotypes induce a normal cell to transform into a precancerous lesion, which subsequently becomes an invasive lesion (Kasi et al., 2021). HPV infection causes the overexpression of viral oncogenes, which may inhibit a number of cellular proteins and affect biological processes such as cell proliferation, cell cycle, and apoptosis (Martínez-Rodríguez et al., 2021).

Globally, in 2020, a total of 604127 cases of cervical cancer were estimated, and 341831 deaths were recorded (Olthof et al., 2024). The incidence rate was 13.3 cases per 100,000 women, and the mortality rate was 7.2 deaths per 100,000 women (Singh et al., 2023). Over 58% of cervical cancer cases globally were estimated in Asia, followed by Africa (20%), Europe (10%) and Latin America (10%) (Singh et al., 2023). However, the incidence and mortality rates for cervical cancer are exceptionally high in Sub-Saharan Africa at 19.59% and 24.55% annual global burden, respectively (Black and Richmond, 2018).

Botanicals’ secondary metabolites and their derivatives have been evaluated in oncology-related clinical research and trials (Omara et al., 2022). The comprehensive knowledge on the mechanism of action for some investigated plant compounds provided the basis to develop novel, efficacious, safer botanical-derived compounds or their semi-synthetic analogues. Such had additional therapeutic advantages over the convectional chemotherapeutic drugs, which are associated with high toxicity not only to cancer cells but also to noncancerous cells (Scatchard et al., 2012; Vordermark, 2016; Johnson et al., 2018; Paken et al., 2023). Cervical cancer has become resistant to conventional therapeutic drugs, including cisplatin, paclitaxel, and carboplatin (Small et al., 2017), thus rendering them less effective. Hence, it was imperative to speed up research on botanical plants as an alternative source of less toxic, more tolerable and effective anticancer drugs for cervical cancer treatment.

The rich biodiversity has contributed significantly to traditional medicine and medicinal systems since ancient times (Omara, 2020). Approximately 1,200 medicinal plants are used in Kenya, 41 of which have anticancer properties, which account for only 10% of the medicinal plants (Onyancha et al., 2018). Botanical-based drugs are gaining attention due to their low toxicity, availability, cost-effectiveness, and tolerability compared to conventional drugs (Tariq et al., 2017; Ochwang’i et al., 2014; Misonge et al., 2016). In this study, *Launaea cornuta* (Hochst. ex Oliv. & Hiern) C. Jeffrey also known as “mchunga” was investigated. *L. cornuta* is an erect herbaceous perennial plant belonging to the Asteraceae family, characterised by a hollow leafy and stem, slightly succulent, glabrous, with milky juice and grows to a height of about 1.5 m (J et al., 2015). It is indigenous to Kenya, Burundi, Cameroon, Uganda, Nigeria, Rwanda, Chad, Djibouti, Eritrea, Ethiopia, Malawi, Mozambique, Somalia, Sudan, Tanzania, Zambia, Central African Republic, Zaïre, and Zimbabwe (Machocho et al., 2014; Omara et al., 2022). In Kenyan communities, this plant is used as a wild vegetable and a source of vitamin C (Musila et al., 2013). The localised budding root concoction known as ‘Kipche’ is used to cure throat cancer (“Koroitab mokto”), typhoid, gonorrhoea, benign prostate hyperplasia, and breast cancer, among others (J et al., 2015; Fatuma Some, 2014; Khan et al., 2016; Chemweno et al., 2022). Regardless of the extensive use of *L. cornuta* plants in traditional medicine, their therapeutic efficacy, and toxicity have not been empirically validated, especially in cervical cancer.

In this study, we systemically evaluated the anticancer activity of the ethyl acetate fraction of *L. cornuta*, analysed and identified compounds using the GC-MS approach and explored the relevant targets and pathways involved in eliciting the anticervical effects through a network analysis approach.

## Materials and methods

### Samples collection and extract preparation

Fresh stems and leaves of *L. cornuta* (Hochst. ex Oliv. & Hiern) C. Jeffrey were collected from Embu County, Mbeere South sub-County, Mavuria ward (Latitude 0°46′27.0″S 37°40′54.9″E and Longitude −0.774156, 37.681908). The plant was not classified as an endangered species, and therefore, no prior permission/consent was sought during the plant material collection phase. Plant identification and authentication were performed by a botanist at the University of Nairobi, and a voucher specimen number NSN18 was given and deposited at the Kenyan National Museums - Eastern Africa Herbarium. The stems and leaves were washed under running tap water, dried under a shade for 3 weeks and milled into a fine powder using an electric grinder (Christy 8 MILL, serial No; 51474). The plant powder was extracted using dichloromethane: methanol solvent following a method described by Okpako et al. with some modifications to obtain the crude extract (Okpako et al., 2023a) and three fractions: n-hexane, ethyl acetate, and water were obtained. The crude extract obtained was macerated in 250 mL of n-hexane solvent, shaken vigorously and left undisturbed for about 20 min. Thereafter, the hexane fraction was separated. Another 250 mL was added to the residues from the first fractionation, and the process was repeated after the two hexane fractions were pooled together. The resulting residues were further fractionated with water and ethyl acetate solvents. The resulting mixtures with intermittent shaking for 30 min were left to stand for 24h and were separated based on the solvent interphase. The organic solvent fractions were concentrated with a rotor evaporator, as described before (Okpako et al., 2023a). The crude extract and the fraction of hexane, water and ethyl acetate were stored at −20°C until analysis.

### Cell lines and culture conditions

A human cervical cancer cell line (HeLa-229) and a kidney epithelial cell line derived from the African green monkey (Vero-CCL) were purchased from ATCC and cultured in Essential Modified Eagle Medium (EMEM) supplemented with 1% L-glutamine (200 mM), 10% fetal bovine serum (FBS), 1.5% sodium bicarbonate, 1% HEPES (1M), 1% penicillin-streptomycin and 0.25 μg/mL amphotericin B. The cell culture was conducted at 37^°^C under a humidified atmosphere and 5% CO_2_ to achieve 75% – 80% confluence.

### Antiproliferative and cytotoxicity assay

The effects of *L. cornuta* fractions and crude extract on the proliferation of HeLa cells were evaluated using the 3-(4, 5-dimethylthiazol-2-yl)-2, 5-diphenyltetrazolium bromide (MTT) assay (Solarbio, China) following a method described by Nyeru *et al.* and Sergazy *et al.* with some modification (Njeru and Muema, 2021; Sergazy et al., 2021). The HeLa-229 cells (1.0 × 10^4^ cells/mL) were seeded in a 96-well plate containing fresh EMEM medium and incubated for 24 h. Subsequently, the medium was aspirated, and 100 µL of 200 μg/mL of the plant fractions and the total extract were added and incubated for 48 h. Doxorubicin (200 μg/mL) was used as a positive control, and 0.2% dimethyl sulfoxide was used as a negative control. After incubation for 48 h, 10 µL of freshly prepared 5 μg/mL MTT was added to each well and incubated for 4 h. MTT was then aspirated, and 100 µL of 100% dimethyl sulfoxide (DMSO, Finar Chemicals, India) was added to dissolve formazan crystals. The absorbance was read at 570 and 720 nm using a plate reader (Infinite M1000 by Tecan). All experiments were carried out in four replicates, and the percentage cell viability was calculated using the formula presented below (Juwitaningsih et al., 2022).

$$\text{Cell viability }\left(\%\right)=\left[\left(\text{Absorbance of treated cell }–\text{ Absorbance of culture medium}\right)/\left(\text{Absorbance of untreated cell }–\text{ Absorbance of culture medium}\right)\;x\;100\right]$$

The *L. cornuta* ethyl acetate fraction, which gave less than 50% cell viability after 48 h of incubation, was considered to have anti-cervical cancer activity and was prioritised for further downstream investigations. Thereby, ethyl acetate fraction was selected for dose-dependent tests using a range of concentrations (2.0–64.0 μg/mL) and (4.0–256 μg/mL) for the cytotoxic effect on HeLa-229 and Vero-CCL cells, respectively. The half-maximal inhibitory concentration (IC_50_) and the half-maximal cytotoxicity concentration (CC_50_) were determined using dose-response and dose-cytotoxicity graphs and calculated by a non-linear regression method using GraphPad Prism 8.4 (Panyajai et al., 2024). The selectivity index (SI) was calculated using the formula below (Mongalo et al., 2017).

$$\text{Selectivity Index }\left(\text{SI}\right)=\left[\left({\text{CC}}_{50}\text{ in }µg/\text{mL}\right)/\left({\text{IC}}_{50}\text{ in }µg/\text{mL}\right)\right]$$

### Cell morphological study

To investigate the effects of *L. cornuta* ethyl acetate on HeLa 229 cells, the cells were treated with five different concentrations of the extract (4, 8, 16, 32, and 64 μg/mL) for a period of 48 h. The morphological changes in HeLa cells were then determined with a digital microscope at ×20 magnification.

### Wound healing assay

HeLa 229 cells were cultured in a 6-well plate at a density of 1 × 10^6^ cells/mL per well and incubated for 24 h to form a monolayer. A wound was created using a sterile 200 mL pipette tip. The cells were then treated with *L. cornuta* ethyl acetate fraction at the concentration corresponding to the IC_50_ (20.56 μg/mL) and incubated for 48 h. During the treatment period, cells were imaged using a digital microscope at 0 h, 24 h and 48 h. The distance between the wounds on the cells was measured and analysed with ImageJ software. The relative migration distance was calculated and expressed as a percentage, as shown below.

$$\text{Relative migration distance }\left(\%\right)=\left[\left(\text{Distance within the scratch at }0\;h\;–\text{ Distance within the scratch at }24\;h)/(\text{Distance within the scratch at }0\;h\right)x\;100\right]$$

### Preliminary screening and phytochemical analysis using GC-MS

We performed a preliminary screening for the presence of alkaloids, glycosides phenols, flavonoids, terpenoids, steroids, quinones, saponins, and tannins in *L. cornuta* ethyl acetate fraction, which exhibited anti-cancer activity using methods described previously (Njeru et al., 2015; Yadav et al., 2017; Shaikh and Patil, 2020) and subsequently, gas chromatography-mass spectrometer system (Model; Shimadzu, GC-MS QP-2010SE) was used for quantitative analysis. The system was equipped with a low-polarity BPX5 capillary column (30 m × 0.25 mm × 0.25 μm film thickness). The oven temperature was set at 55°C for 1 min, then increased by 10°C per minute to reach a constant temperature of 280°C for 15 min and held for 30 s. The injector temperature was set at 200°C. Helium was used as the carrier gas with a 1.08 mL/min flow rate. The diluted sample (1% v/v) was injected (1 µL) into the GC with an AS3000 autosampler in a split ratio of 10:1. The mass detector was set at 200°C (ion source) and 250°C (interface temperature). Electron-ionisation mass spectra were collected at 70 eV in full scan mode at a m/z of 35–550. The identity of phytochemicals was determined by comparing the mass spectra with the spectra of compounds in the NIST library database (Wavinya Nyamai et al., 2015; Njeru and Muema, 2020).

### Bioinformatics analysis

#### Drug likeness screening test

The PubChem CID and canonical SMILES (Simplified Molecular Input Line Entry System) of the GC-MS identified compounds of *L. cornuta* ethyl acetate were retrieved from PubChem (https://pubchem.ncbi.nlm.nih.gov/). The SMILES were then submitted to SwissADME and pkCSM tools (http://www.swissadme.ch/index.php & https://biosig.lab.uq.edu.au/pkcsm/prediction) to predict the druglike (DL), the pharmacokinetics and physicochemical properties of the compounds. The prediction was based on Lipinski’s rule of five: blood-brain barrier (BBB), the central nervous system (CNS), human intestinal absorption, and inhibition of cytochrome P450s (Vishvakarma et al., 2023).

### Identification of candidate targets for *Launaea cornuta* ethyl acetate fraction against human cervical cancer

The targets of the compounds were predicted from Swiss TargetPrediction (http://www.swisstargetprediction.ch/database), with ‘humans’ (*Homo sapiens*) as the study species with *p* > 0.1 considered potential targets (Daina et al., 2019). Additional targets were predicted using BindingDB (https://bindingdb.org/rwd/bind/chemsearch/marvin/FMCT.jsp), potential targets with *p* > 0.7 were considered and retrieved. The Gene IDs of the selected targets were retrieved from UniProtKB database (https://www.uniprot.org). Ensemble approach (SEA) (https://sea.bkslab.org/) database was used to predict the compound’s targets based on the assumption that molecules with similar structures tend to have similar responses to a target (Wang et al., 2016). For each database, the term ‘*H. sapiens*’ was used as the keyword. The results predicted from the three databases were pooled, and duplicates were removed (Li and Ren, 2022). The disease targets of human cervical cancer for development and progression were retrieved from GeneCards (https://www.genecards.org/), DisGeNET (https://www.disgenet.org/), OMIM (https://www.omim.org/) and Pharos (https://pharos.nih.gov/diseases/) databases using ‘Cervical cancer’ the disease type. All retrieved targets were merged, and only the unique targets were selected. Lastly, the potential targets of the identified compounds of *L. cornuta* ethyl acetate and the disease targets of cervical cancer were intersected using an online bioinformatics and evolutionary genomics platform (https://bioinformatics.psb.ugent.be/webtools/ven) to obtain the common target genes.

### Protein-protein interaction (PPI) analysis

The STRING database (https://string-db.org/), version 12.0, was used to build a PPI network for the common target genes between the *L. cornuta* ethyl acetate fraction and cervical cancer targets. The species was set as “*H. sapiens*”, and the interaction threshold was set to 0.4. Subsequently, the PPI data was exported as a ‘TSV’ format file and imported into Cytoscape software (version 3.10) for analysis. The Cytohubba plug-in in Cytoscape was used to determine and calculate the score (degree) of each node and the top 30 hub targets were selected (Zhang et al., 2021).

### Gene ontology (GO) and Kyoto encyclopaedia of genes and genomes (KEGG) enrichment analyses

GO and KEGG pathway enrichment analysis was performed following the method described by Jeddi et al. with some modifications (Jeddi et al., 2023) for the common targets of between *L. cornuta* ethyl acetate fraction and cervical cancer using an online enrichment tool ShinyGO version 0.77 (https://ge-lab.org/go/), SRplot (Science and Research online plot (bioinformatics.com.cn) tool was used to identify the KEGG pathway and mapping associated with the targets, Human was selected as the species with a false discovery rate FDR cut-off of 0.05, and the number of pathways to be presented was set as 20. The cellular components (CC), molecular functions (MF), biological process (BP) and pathway enrichment terms with a *p*-value of <0.05 were identified as significantly enriched and considered for subsequent analysis.

### Molecular docking

The 3D structures of AKT1 (1H10), MDM2 (5C5A), CDK2 (4GCJ), P53 (7LIN) BCL-2 (6O0O), CASP9 (4RHW) AND P21 (5DEW) were retrieved from the Protein Data Bank (https://www.rcsb.org/). The 3D structures of the *L. cornuta* compounds and the native ligands of the targeted proteins were downloaded from PubChem (https://pubchem.ncbi.nlm.nih.gov/) and converted to the PDBQT (Protein Data Bank, Partial Charge (Q), and Atom Type (T)) format using Open Babel plug-in in the PyRx software. Ligand energy minimisation was performed using the PyRx software. The grid box for each protein was maximised to fully enclose the protein structure, and blind docking was performed using the VINA tool in the PyRx, with an exhaustiveness of 8. The compounds with the binding affinity less than −7.5 kcal/mol were selected and visualised with Discovery Studio 2021 Client. Docking validation was conducted by redocking native ligands to the target proteins.

### Relative gene expression using quantitative polymerase chain reaction (qPCR) assay

In this study, a quantitative RT-PCR assay was conducted to measure the transcript levels of AKT1, TP53, BCL-2, MDM2, CDK2, Casp9, and P21 proteins. First, total RNA was extracted from HeLa cells using the SolarBio Total RNA Extraction Kit (Beijing, China) following the manufacturer’s instructions. Then, the quality of the RNA was assessed using a nanodrop spectrophotometer (Thermofisher). The RNAs (1 µg) were then converted to complementary DNA (cDNA) using the SensiFAST cDNA synthesis kit (Brisbane, Australia) following the manufacturer’s protocol. Quantitative RT-qPCR was performed using the SensiFAST™ SYBER^@^ Lo-ROX Kit (Bioline, Australia). The program for the qPCR machine (QuantStudio 5; Applied Biosynthesis) was set as follows: initial denaturation at 95°C for 2 min, 1 cycle; denaturation at 95°C for 5 s, 40 cycles; annealing at 62°C for 10 s and extension at 72°C for 20 s, 40 cycles and melt curve at 60 s, 1 cycle. GAPDH was used as the reference gene and the relative expression was calculated using the 2^^−ΔΔct^ method (Njeru et al., 2020). The primer sequences used in this RT-qPCR analysis are presented in Supplementary Table S1.

### Statistical analysis

All data described in this study were expressed as mean ± standard deviation (M±SD). GraphPad Prism 8.4 software (San Diego, CA, USA) was used to perform all the statistical analyses. Statistical comparisons were conducted using a one-way analysis of variance (ONE-WAY ANOVA), followed by the Turkey multiple comparison test. The differences were then considered statistically significant at *p <* 0.05.

## Results

### Screening of *Launaea cornuta* extract fractions for antiproliferative activity

**FIGURE 1:**
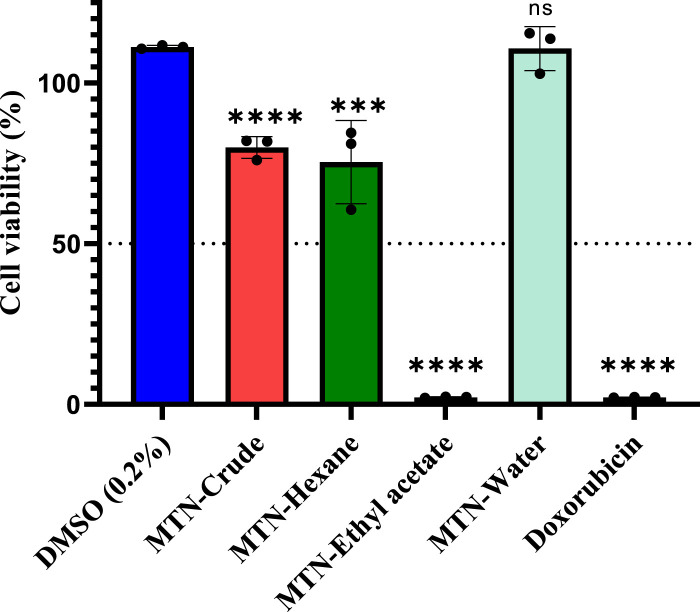
Screening of antiproliferative activity of *Launaea cornuta* extract and its fractions at a fixed concentration of 200 μg/mL on HeLa-229 cell line. Doxorubicin (200 μg/mL) was used as a positive control, and 0.2% DMSO as the negative control. All treatments were carried out in triplicate (*n* = 3). Statistical significance was calculated by nonlinear regression compared to the negative control (DMSO, 0.2%) and *, *p* < 0.05; **, *p* < 0.01, ***, *p* < 0.001 and ****, *p* < 0.0001. DMSO represents dimethyl sulfoxide; MTN represents *Launaea cornuta.*

We performed MTT assay to evaluate the antiproliferative activity of the crude *L. cornuta* extract and its solvent fractions (hexane, ethyl acetate and water fractions) at a fixed concentration (200 μg/mL) against human cervical cancer cell lines. The ethyl acetate fraction significantly (*p <* 0.0001) exhibited a robust antiproliferative effect on the HeLa cell lines, suppressing cancer cell growth by more than 50% (Figure 1). The crude extract, hexane, had significant antiproliferative effects (*p* < 0.002 and *p* < 0.0001, respectively) but not within our set tight threshold, while water fractions had no activity (*p* > 0.9) compared to the negative control. Based on these criteria, we, therefore, selected the *L. cornuta* ethyl acetate fraction for concentration-dependent tests to obtain the minimum inhibitory concentration (IC_50_).

### Dose-dependent cytotoxicity

**FIGURE 2:**
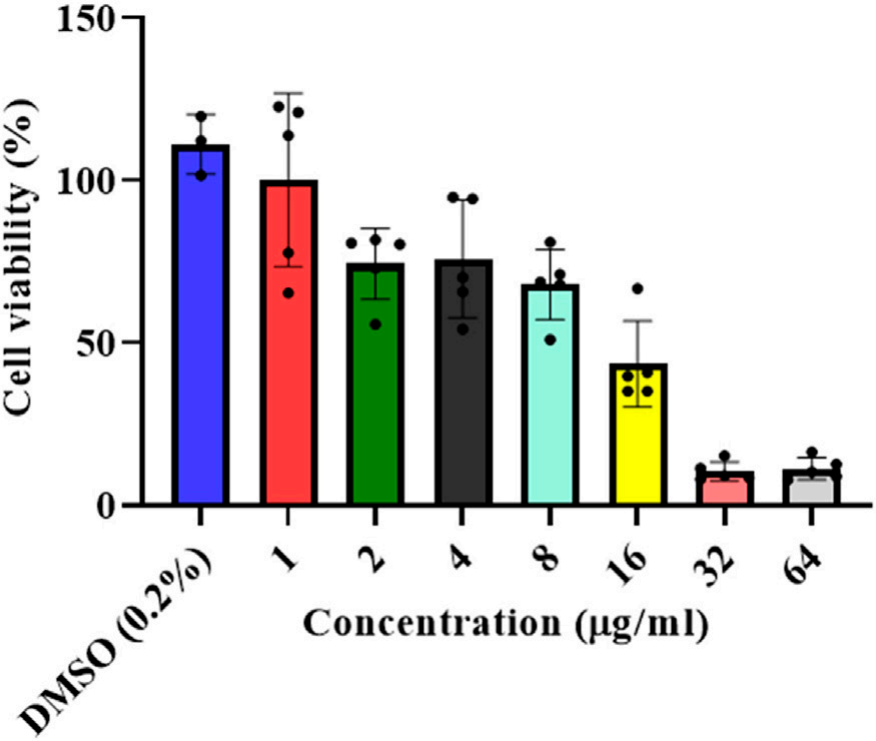
*In vitro* antiproliferative assay at different concentrations of the *Launaea cornuta* ethyl acetate fraction against HeLa cells after 48 h of incubation. Mean ± SD values are expressed independently of three minimum experiments.

**FIGURE 3:**
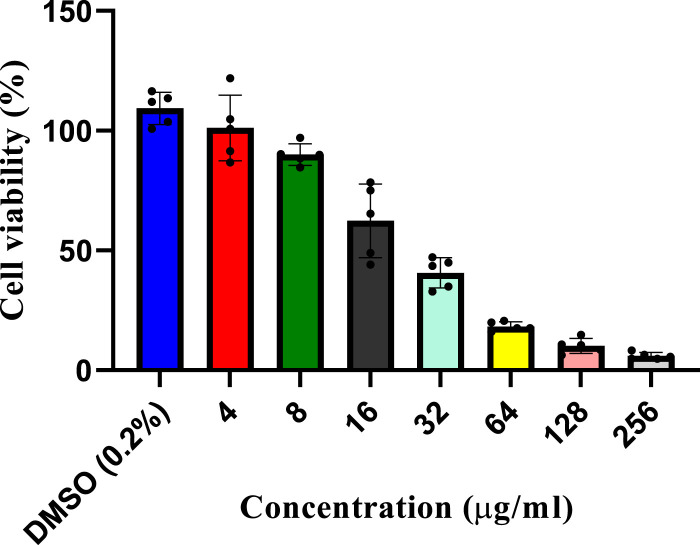
*In vitro* cytotoxicity assay at different concentrations of the *Launaea cornuta* ethyl acetate fraction against Vero cells after 48 h of incubation. Mean ± SD values are expressed independently of three minimum experiments.

A dose-dependent cytotoxicity test was performed to determine the concentration of the *L. cornuta* ethyl acetate fraction that selectively inhibited the growth of Hela cells by determining both IC_50_ and CC_50_) (Figures 2, 3). HeLa cells and Vero cells were treated with ethyl acetate fraction of *L cornuta* at concentrations ranging from 1–64 μg/mL (HeLa cells) and 4–256 μg/mL (Vero cells). After 48 h, *L. cornuta* ethyl acetate IC_50_ and CC_50_ for HeLa and Vero cells were determined as 20.56 ± 2 μg/mL and 48.83 ± 23 μg/mL, respectively. Doxorubicin exhibited an IC_50_ of 2.09 ± 1.35 μg/mL on Hela cells and a CC_50_ of 3.44 ± 1.00 μg/mL on Vero cells, respectively (Table 1). The dose-dependent test results for positive control (doxorubicin) are presented in the Supplementary Figure S1.

**TABLE 1:** Selectivity Index (SI) of *Launaea cornuta* ethyl acetate fraction and doxorubicin.

Extract	IC_50_ (µg/mL)	CC_50_ (µg/mL)	SI
*L. cornuta* ethyl acetate fraction	20.56 ± 2.83	48.83 ± 23.02	2.36
Doxorubicin	2.09 ± 1.35	3.44 ± 1.00	1.64

**FIGURE 4:**
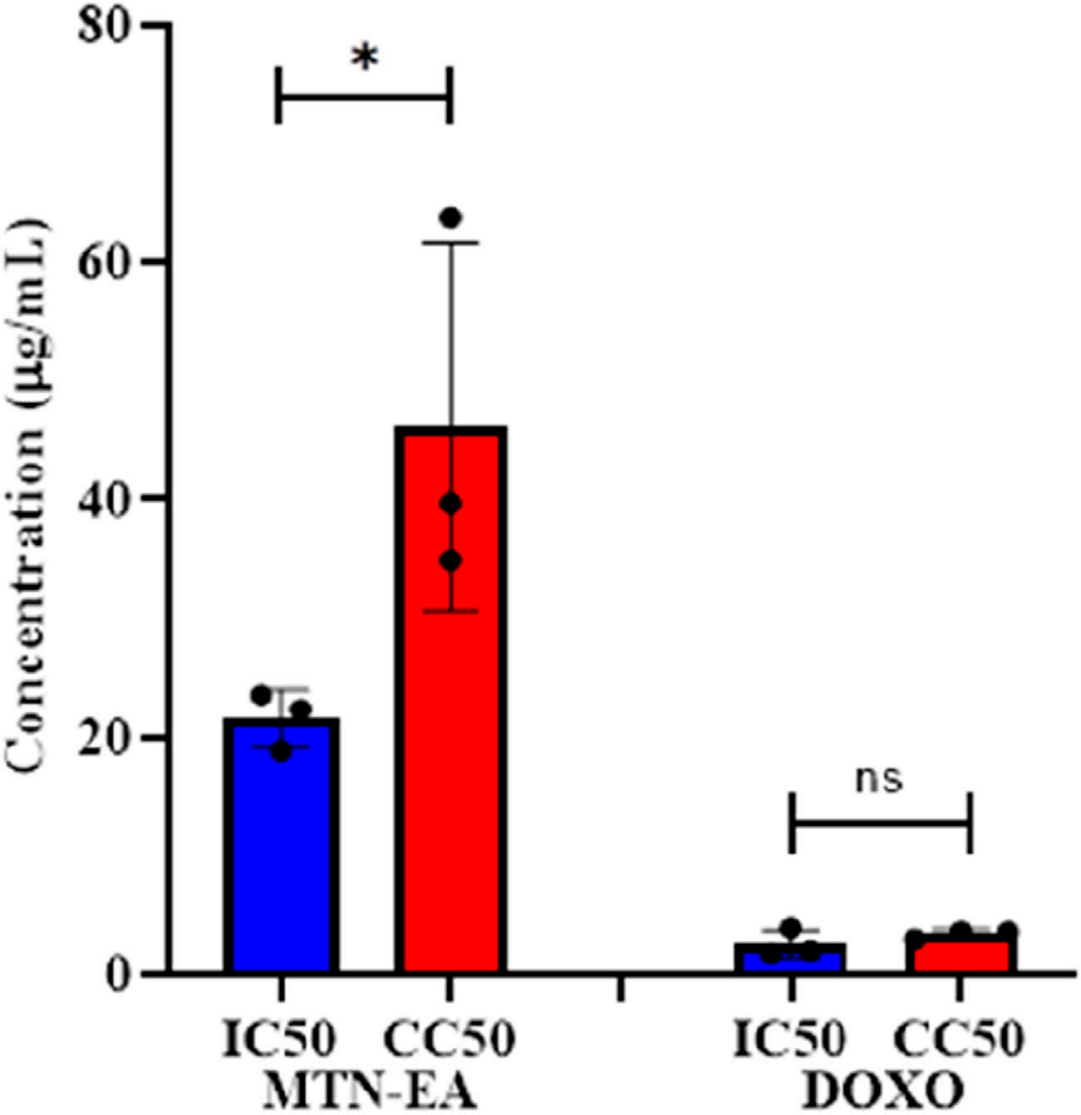
Half-maximal concentration and half-maximal cytotoxic concentration of *Launaea cornuta* ethyl acetate and doxorubicin on HeLa and Vero cells after 48 h of incubation. There is a significant difference between IC_50_ and CC_50_ values (*p* < 0.01). Mean ± SD values are expressed independently of three minimum experiments.

Selectivity index (SI) was calculated to assess the selective toxicity of *L. cornuta* ethyl acetate fraction toward cervical cancer cells compared to normal cells. The resulting SI value for the HeLa cell was 2.37 and 1.64 for *L. cornuta* ethyl acetate fraction and doxorubicin, respectively (Table 1; Figure 4), demonstrating high selectivity of *L. cornuta* ethyl acetate fraction to cancer cells.

### Morphological study

**FIGURE 5:**
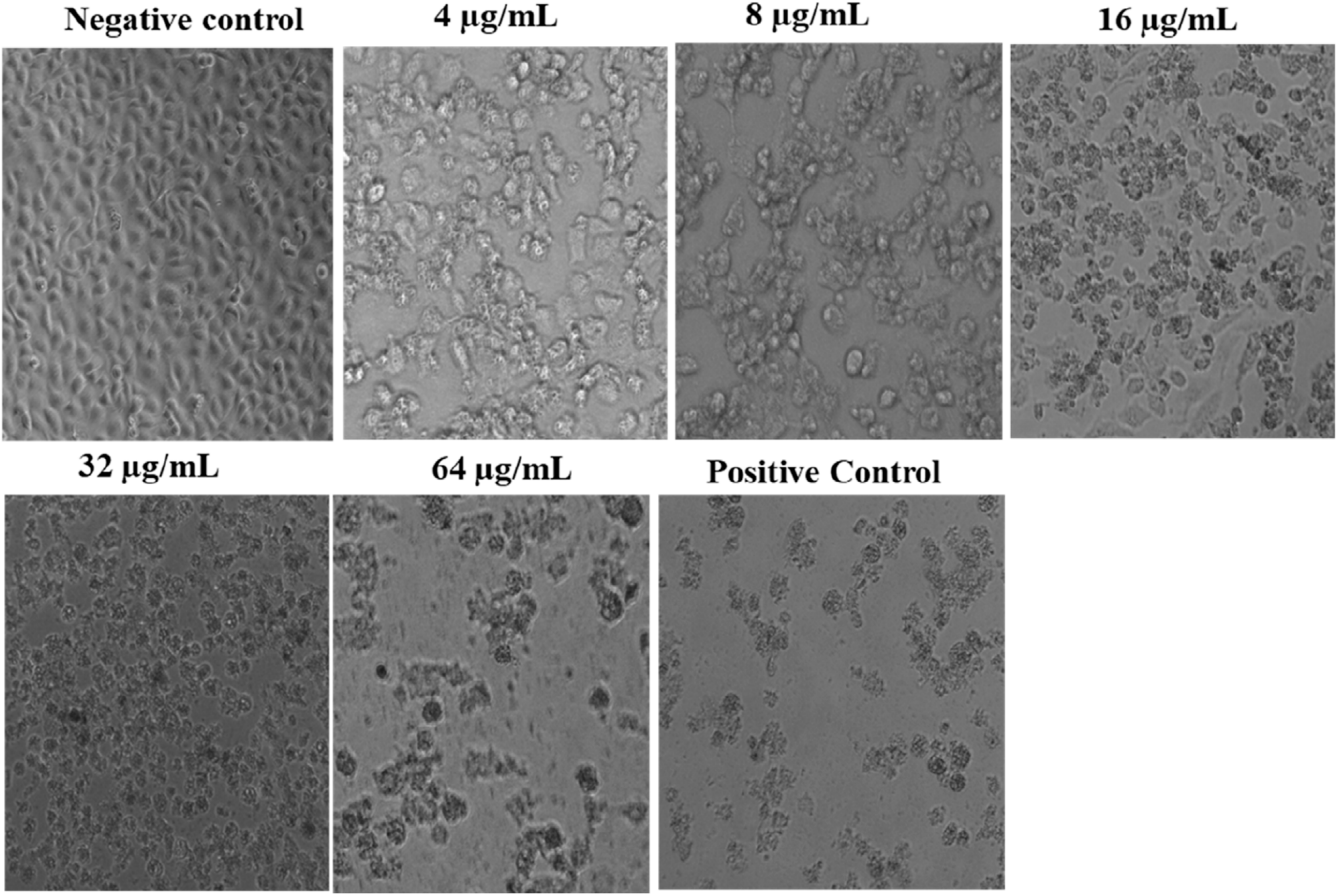
Morphological changes in HeLa cells after exposure to varying concentrations of *Launaea cornuta* ethyl acetate fraction over 48 h. (magnification ×15). Negative control; 0.2% DMSO and Positive control; Doxorubicin drug (2.09 μg/mL).

We confirmed the effect of *L. cornuta* ethyl acetate fraction on the morphology of HeLa cells for 48 h under a digital microscope. HeLa cells exposed to varying concentrations of *L. cornuta* ethyl acetate fraction exhibited atypical morphology with cellular shrinkage, sphere-shaped and surface detachment, indicating that the extract fraction had cytotoxic effects (Figure 5) as compared to the negative control. Notably, the effects of the extract fraction was dose-dependent.

### Effect of *Launaea cornuta* ethyl acetate on cell migration

**FIGURE 6:**
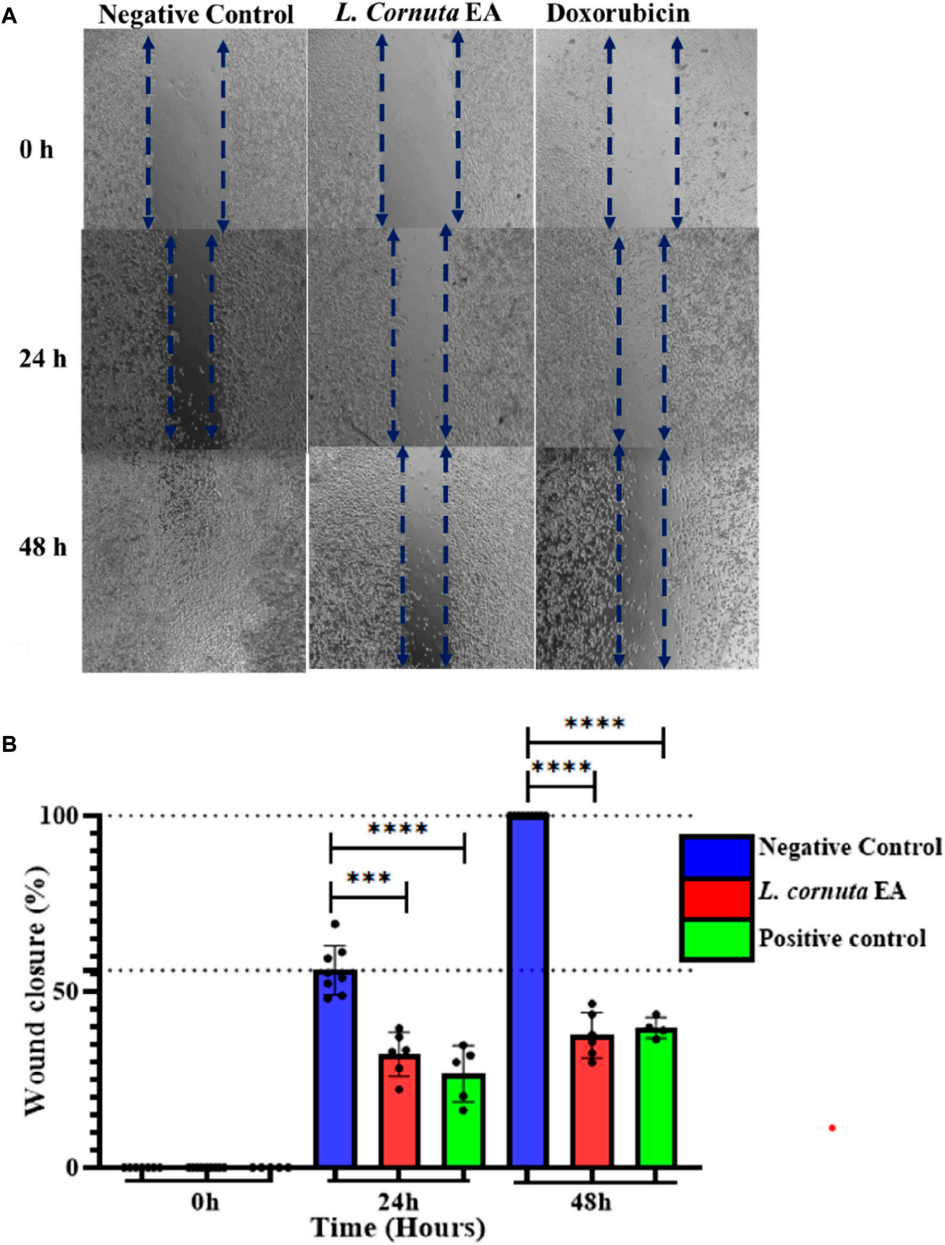
*Launaea cornuta* ethyl acetate inhibited HeLa cells migration ability after treatment at a concentration of 20.56 μg/mL (IC_50_). Images were obtained at time points of 0, 24, and 48 h were taken to capture images. **(A)** photomicrographs show the anti-migration effects of *Launaea cornuta* ethyl acetate fraction on HeLa cells as compared with negative control. **(B)** Percentage of wound healing area. Each bar graph shows the wound closure (%) of HeLa cells. Wound areas were measured at each time point and expressed as a percentage of reduction area in comparison with 0 h of incubation. The percentages of wound closure were statistically compared to the negative control. Each bar represents mean ± SD of at least three independent experiments performed in triplicate. ***, *p* < 0.001 and ****, *p* < 0.0001.

A wound-healing assay was conducted to investigate the effect of *L. cornuta* ethyl acetate fraction on cancer cell migration. The results indicated a decrease in wound size in the control cells (DMSO, 0.2%), which eventually closed after 48 h compared with treated HeLa cells. In contrast, the HeLa cells treated with *L. cornuta* ethyl acetate fraction at IC_50_ of 20.56 μg/mL inhibited wound closure. The results indicated that *L. cornuta* ethyl acetate significantly (*p* < 0.0001) inhibited cell migration compared to the negative control after 24 and 48 h. However, there were no significant differences between *L. cornuta* ethyl acetate and the positive control at 24 and 48 h (*p* > 0.6 and *p* > 0.9, respectively). The substantial decrease in migration and minimum closure in wound size was attributed to the increased cell mortality and/or inhibition of cell migration mediated by the extract fraction (Figure 6A). The percentage of wound closure for *L. cornuta* ethyl acetate fraction (20.56 μg/mL) was 35.9% and 41.9% for 24 and 48 h (Figure 6B).

### Preliminary screening and gas chromatography-mass spectrometry (GC-MS) analysis of phytochemicals in *L. cornuta* ethyl acetate

**FIGURE 7:**
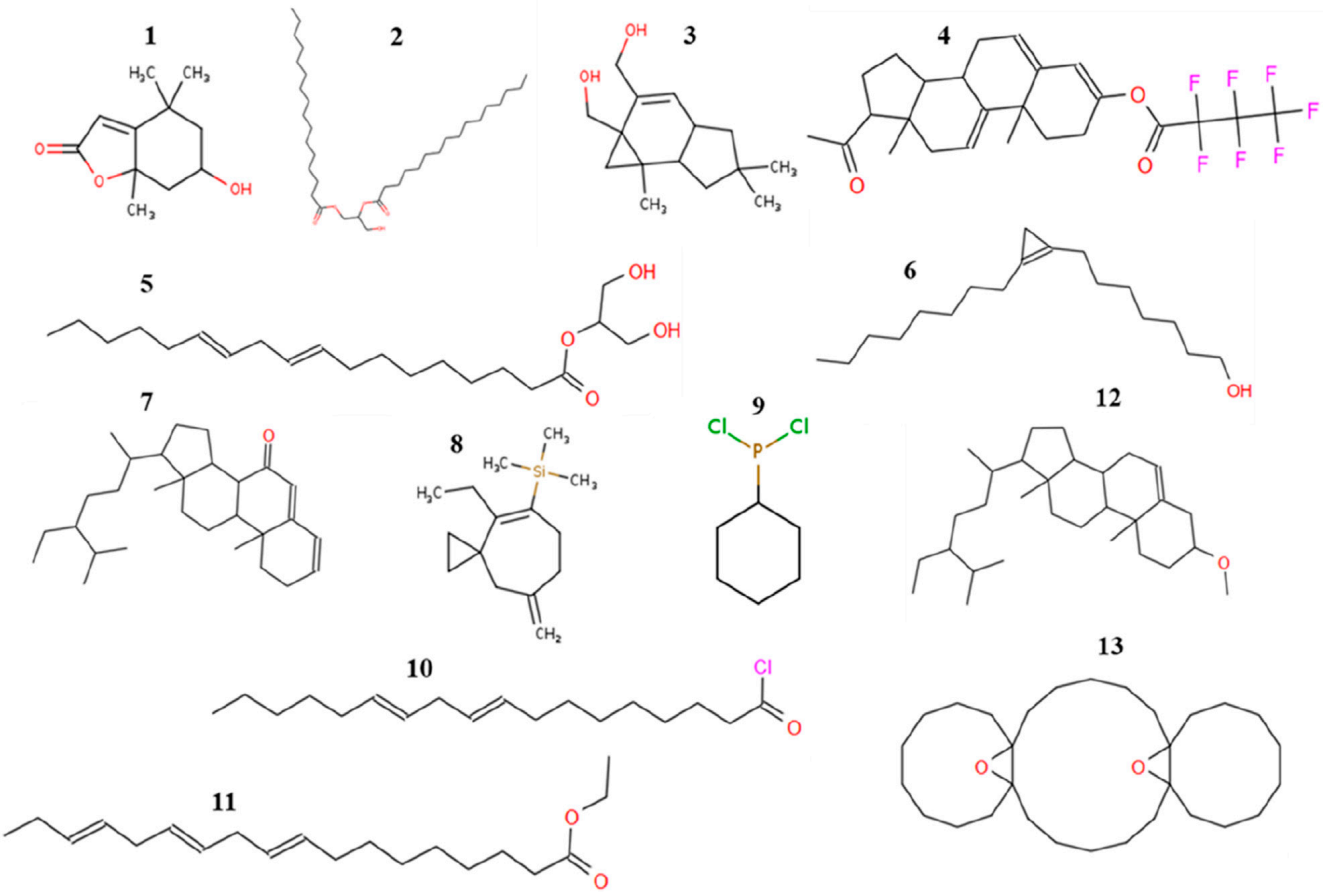
Numbers 1–13 showed the 2D structures of the compounds identified in *Launaea cornuta* ethyl acetate fraction (Details refer to Table 2).

Having demonstrated selective antiproliferative activity of *L. cornuta* ethyl acetate, we next then identified the phytochemicals that could be attributed to the established antiproliferative activity of the extract fraction. The qualitative phytochemical screening showed broad groups of phytochemicals as follows: glycosides, phenols, saponins, terpenoids, quinones, and tannins (Supplementary Table S2). GC-MS analysis was performed to reveal the semi-quantitative presence of phytochemical profiles. A total of 13 compounds were identified in *L. cornuta* ethyl acetate fraction through GC-MS analysis (Table 2), and the chromatogram showing the 13 peaks is presented in the Supplementary Figure S2. The compounds identified included (9Z,12Z) -octadeca-9,12-dienoyl chloride (57.26%), 2-Octylcyclopropene-1-heptanol (14.48%), Tricyclo [20.8.0.0 (7,16)] triacontane, 1 (22) 7, 7 (16) -diepoxy, Tricyclo [20.8.0.0 (7,16)] triacontane, 1 (22) 7, (16)-diepoxy (7.39%), ethyl (9Z,12Z,15Z)-octadeca-9,12,15-trienoate (4.28%), Cycloprop [e]indene-1a,2(1H)-dimethanol, 3a,4,5,6,6a,6b-hexahydro-5,5,6b-trimethyl-, (1a.alpha,3a.beta,6a.beta,6b.alpha) (2.94%), 6-Hydroxy-4,4,7a-trimethyl-5,6,7,7a-tetrahydrobenzofuran-2(4H)-one (2.73%) and 2-Linoleoylglycerol (2.28%). A previous study reported that 6-Hydroxy-4,4,7a-trimethyl-5,6,7,7a-tetrahydrobenzofuran-2(4H)-one exhibited anti-inflammatory effect against lipopolysaccharide (LPS)-induced raw macrophage (Jayawardena et al., 2019), Glycerol 1,2-dipalmitate possess ant-fungal activity (Sanni and Omotoyinbo, 2016). Ethyl (9Z,12Z,15Z)-octadeca-9,12,15-trienoate exhibited anticancer activity against oral epidermoid carcinoma, breast, colon, and lung cancer (Huang et al., 2022), Stigmast-5-ene, 3beta-methoxy- induced a reduction of cell viability by apoptotic cell death through cell cycle arrest at the sub-G1 stage (Alvarez-sala et al., 2019; Jaiganesh et al.) and tremulone exhibited anticancer activity against cervical, colorectal and breast cancers (Lu et al., 2012; Suliman and El-hddad, 2023). However, most of the compounds identified in this study have not been reported to have biological activity, therefore, further investigation is necessary to evaluate the potential biological activity of each compound. The 2D structures of these compounds are shown in Figure 7.

**TABLE 2:** The phytochemical profile of the *Launaea cornuta* ethyl acetate fraction detected by the GC-MS technique.

Peak no.	RT (minute)	Compound	MF	MW (g/mol)	Relative abundance (%)	Type of compound
1	16.476	6-Hydroxy-4,4,7a-trimethyl-5,6,7,7a-tetrahydrobenzofuran-2(4H)-one	C_11_H_16_O_3_	196.2	2.73	Benzenoids
2	21.249	Glycerol 1,2-dipalmitate	C_35_H_68_O_5_	568.9	1.57	Fatty acid methyl esters
3	21.955	Cycloprop [e]indene-1a,2(1H)-dimethanol, 3a,4,5,6,6a,6b-hexahydro-5,5,6b-trimethyl-, (1a. alpha.,3a.beta.,6a.beta.,6b.alpha.)-(−)-	C_15_H_24_O_2_	236.4	2.94	Terpenoids
4	22.006	3-Heptafluorobutyriloxy-3,5,10-pregnatrien-20-one	C_25_H_27_F_7_O_3_	505.8	1.21	Aliphatic compound
5	22.361	2-Linoleoylglycerol	C_21_H_38_O_4_	354.5	2.28	Glycerolipids
6	22.791	2-Octylcyclopropene-1-heptanol	C_18_H_34_O	266.5	14.48	Fatty alcohols
7	23.798	Tremulone	C_29_H_46_O	410.7	1.26	Terpenoids
8	24.799	Spiro [2.6]non-4-ene, 4-ethyl-8-methylene-5-trimethylsilyl	C_15_H_26_Si	234.5	1.98	Alkenes
9	26.127	Cyclohexyldichlorophosphine	C_6_H_11_Cl_2_P	185.0	0.96	Fatty acids
10	26.127	(9Z,12Z)-octadeca-9,12-dienoyl chloride	C_18_H_31_ClO	298.9	57.26	Fatty acids
11	27.018	Ethyl (9Z,12Z,15Z)-octadeca-9,12,15-trienoate	C_20_H_34_O_2_	306.5	4.28	Fatty acids
12	29.624	Stigmasteryl methyl ether	C_30_H_52_O	428.7	1.67	Phytosterols
13	31.846	Tricyclo [20.8.0.0 (7,16)]triacontane, 1(22),7(16)-diepoxy	C_30_H_52_O_2_	444.7	7.39	Fatty acids

Key: RT, retention time (minute); MF, molecular formula; MW, molecular weight (g/mol).

## *In-silico* results

### Screening for drug-like compounds in *Launaea cornuta* ethyl acetate fraction

**FIGURE 8:**
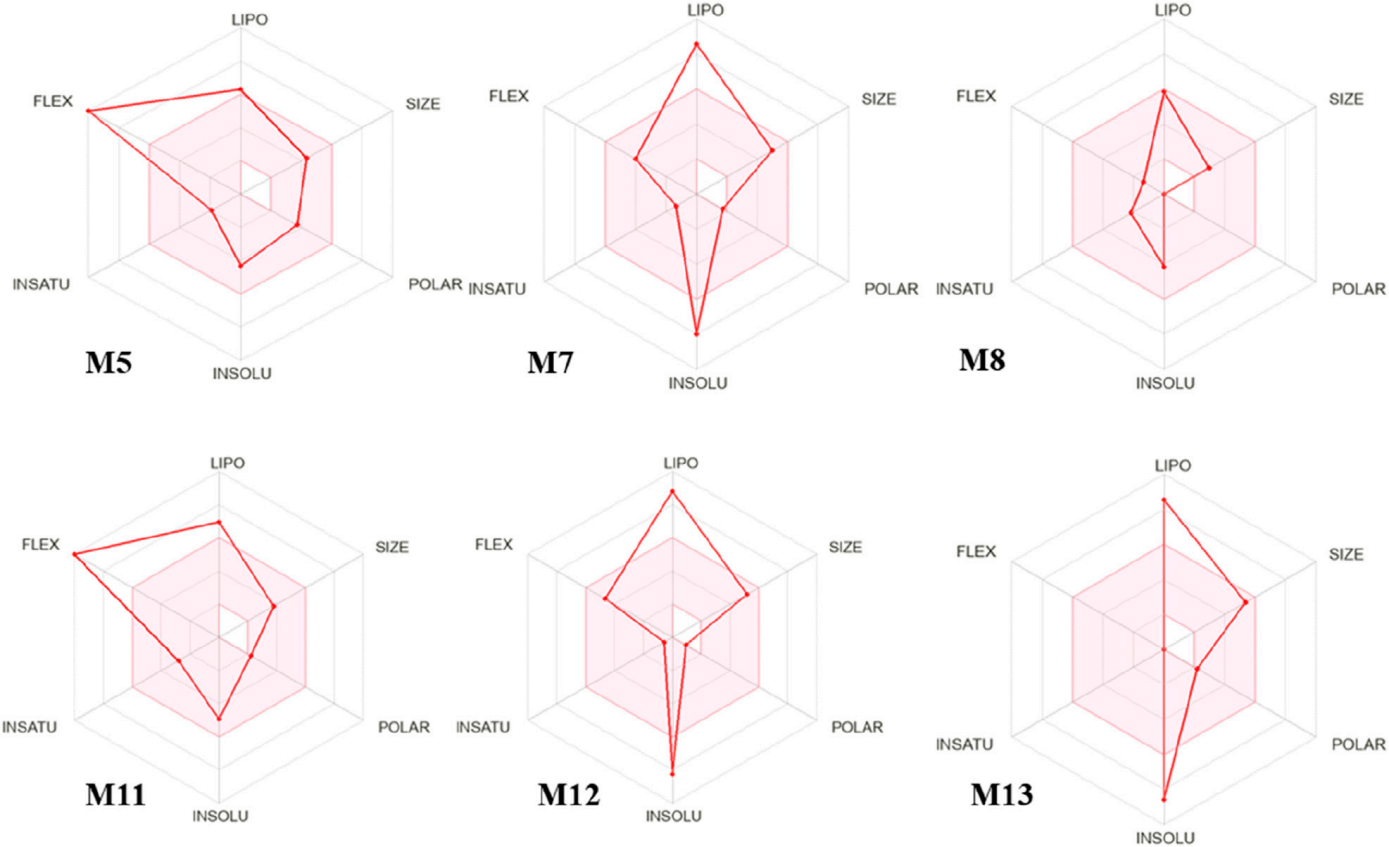
Bioavailability radars of 6 *Launaea cornuta* ethyl acetate fraction compounds, based on the six ideal physicochemical properties for oral bioavailability, namely, polarity (POLAR), lipophilicity (LIPO), saturation (INSATU), size (SIZE), flexibility (FLEX), and solubility (INSOLU).

The chemical compounds of *L. cornuta* ethyl acetate fraction obtained from GC-MS analysis were used in the *in silico* studies. Thirteen compounds were subjected to screening, and their putative pharmacokinetics and physicochemical properties (absorption, distribution, metabolism, excretion and toxicity) were determined using SwissADME and pkCSM databases. Only 6 met the Lipinski’s rule of 5 (RO5) of MW; <500 g/mol, Log P; <5, HBA<10, HBD<5, and RB < 10. In addition to Lipinski’s rules, the compounds were also predicted to be water-soluble and did not interfere with the blood-brain barrier and central nervous system. Furthermore, the ADMET profile suggest that the 6 compounds do not interfere with metabolism, thus minimising the risk of drug-drug interactions (Table 3, Supplementary Table S3). Oral bioavailability was evaluated for the six (6) compounds using a bioavailability radar (Ahmed Khan et al., 2023) (Figure 8). The bioavailability radars were based on six ideally adapted physicochemical properties for oral bioavailability, namely, lipophilicity, polarity, size, solubility, saturation, and flexibility. The thresholds were as follows: lipophilicity (LIPO): 0.7 < XLOGP3 < +5, SIZE: 150 < MV < 500 g/mol, flexibility (FLEX): 0 < Number of rotatable bonds <9, saturation (INSATU): 0.25 < Fraction Csp3 < 1, insolubility (INSOLU): 6 < LOG S < 0, and polarity (POLAR): 20 Å2 < TPSA < 130 Å2. All candidate molecules exhibited oral bioavailability because they were within the pink zone of bioavailability radars. The pink-coloured area presents the fit physicochemical space for oral bioavailability. The graph for each molecule must be adjusted to suit drug-like properties. The prediction of ADMET pharmacokinetic properties demonstrated that all six compounds exhibited good absorption properties as their human intestinal absorptions (HIAs) exceeded 90%. The inhibitory effect on cytochromes indicated that most of them did not affect cytochromes except M5, M7, M11 and M12, which inhibited 3A4, whereas M7, M11, and M12 inhibited 1A2 (Table 3). The ADMES toxicity confirmed that the six drug candidates were not toxic inhibitors. M5, M8, and M11 were predicated to be sensitive to the skin but had no hepatotoxic effect except M13.

**TABLE 3:** Prediction of ADMET *in silico* pharmacokinetic properties of *Launaea cornuta* ethyl acetate compounds.

Compounds number	Absorption	Distribution		Metabolism							Excretion	Toxicity		
	Intestinal absorption (human)			Substrate		Inhibitor					Total excretion			
		Permeability		Cytochromes										
		BBB	CNS	2D6	3A4	1A2	2C19	2C9	2D6	3A4		Ames toxicity	Hepatotoxicity	Skin irritation
	Numeric (% absorbed)	Numeric (log BB)	Numeric (log PS)	Categorical (yes/No)							Numeric (log mL/minute/kg	Categorical (yes/No)		
M1	95.697	−0.198	−3.36	No	No	No	No	No	No	No	1.038	No	No	Yes
M2	85.807	−0.943	−3.114	No	Yes	No	No	No	No	No	2.171	No	No	No
M3	92.7	0.091	−2.37	No	No	No	No	No	No	No	−0.007	No	No	No
M4	91.346	0.185	−3.244	No	Yes	No	No	No	No	No	−0.103	No	No	No
M5*	90.602	−0.294	−3.227	No	Yes	No	No	No	No	No	2.165	No	No	Yes
M6	90.308	0.837	−1.802	No	Yes	No	No	No	No	No	1.715	No	No	Yes
M7*	97.921	0.827	−1.589	No	Yes	Yes	No	No	No	No	0.571	No	No	No
M8*	93.522	0.763	−2.141	No	No	No	No	No	No	No	1.11	No	No	Yes
M9	90.275	0.684	−1.794	No	No	No	Yes	No	No	No	0.418	No	No	No
M10	91.195	0.81	−1.394	No	Yes	No	No	No	No	No	0.237	No	No	No
M11*	92.747	0.766	−1.509	No	Yes	Yes	No	No	No	No	2.134	No	No	Yes
M12*	96.946	0.903	−4.387	No	Yes	Yes	No	No	No	No	0.663	No	No	No
M13*	91.183	1.466	−3.114	No	Yes	No	No	No	No	No	0.909	No	Yes	No

Key: BBB, Brain-blood barrier; CNS, central nervous system; ADMET, absorption, distribution, metabolism, excretion, and toxicity; * Drug-like compounds (DL).

### Prediction of targets for *Launaea cornuta* ethyl acetate compounds and cervical cancer

**FIGURE 9:**
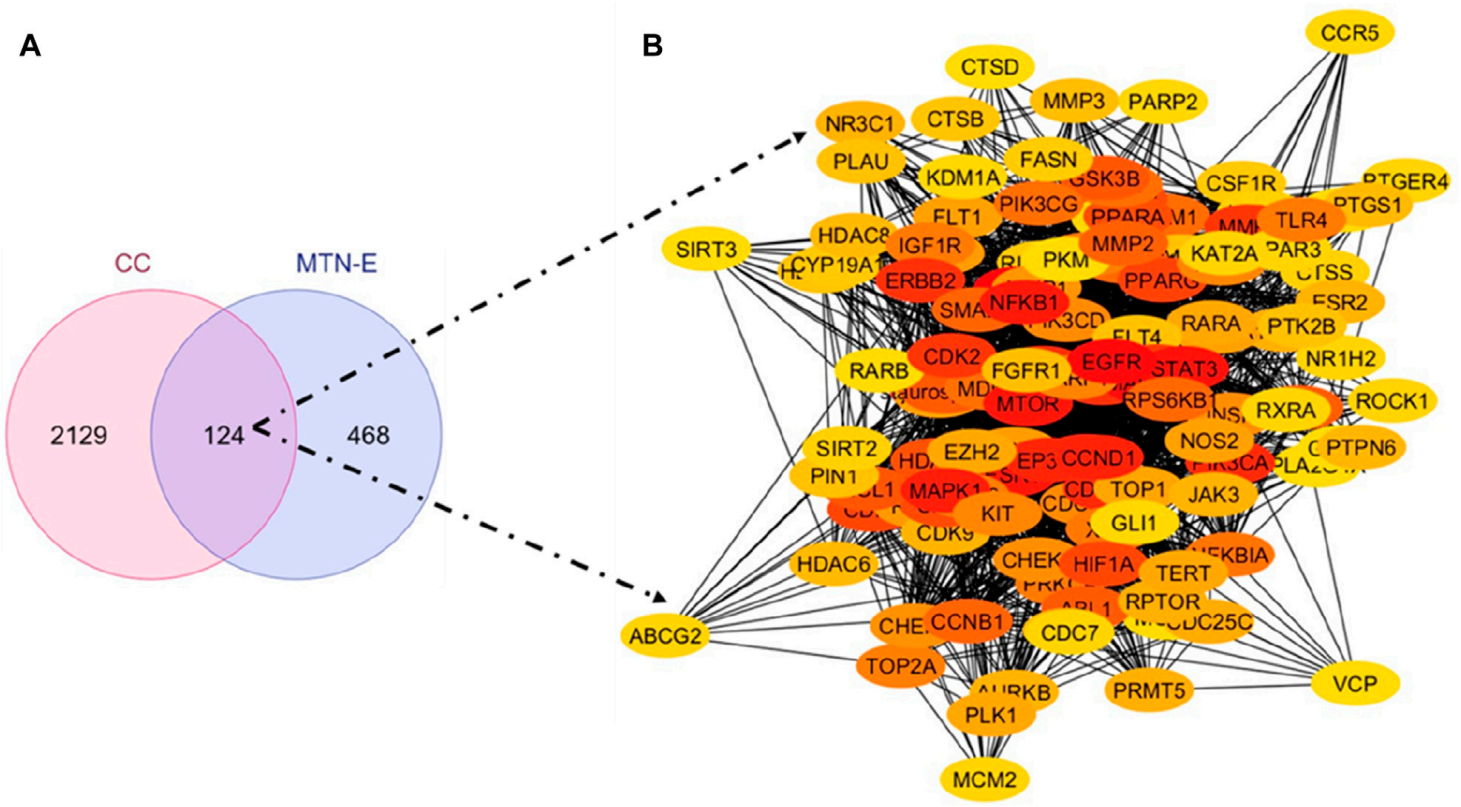
*Launaea cornuta* ethyl acetate-cervical cancer intersection. **(A)** Venn diagram and **(B)** PPI network of the 124 key targets of *Launaea cornuta* ethyl acetate and cervical cancer.

The putative targets of the six (6) compounds of *L. cornuta* ethyl acetate fraction were predicted and retrieved from three databases: SWISS TargetPrediction (STP) with 612 targets, BindingDB (BDB) with 59 targets, and the Similarity Ensemble Approach (SEA) with 503 targets. The targets obtained were merged to obtain 581 unique potential targets. We also predicted human cervical cancer-related genes from 4 databases: GeneCards with 714 targets, DisGeNET with 1817 targets, Pharos with 174 targets, and OMIM with 50 targets to obtain 2253 unique targets. A Venn diagram was generated to show the intersected targets between *L. cornuta* ethyl acetate fraction and cervical cancer as hub genes for subsequent analyses (Figure 9A).

### Construction of protein-protein interaction (PPI) network for the compound-disease targets

**FIGURE 10:**
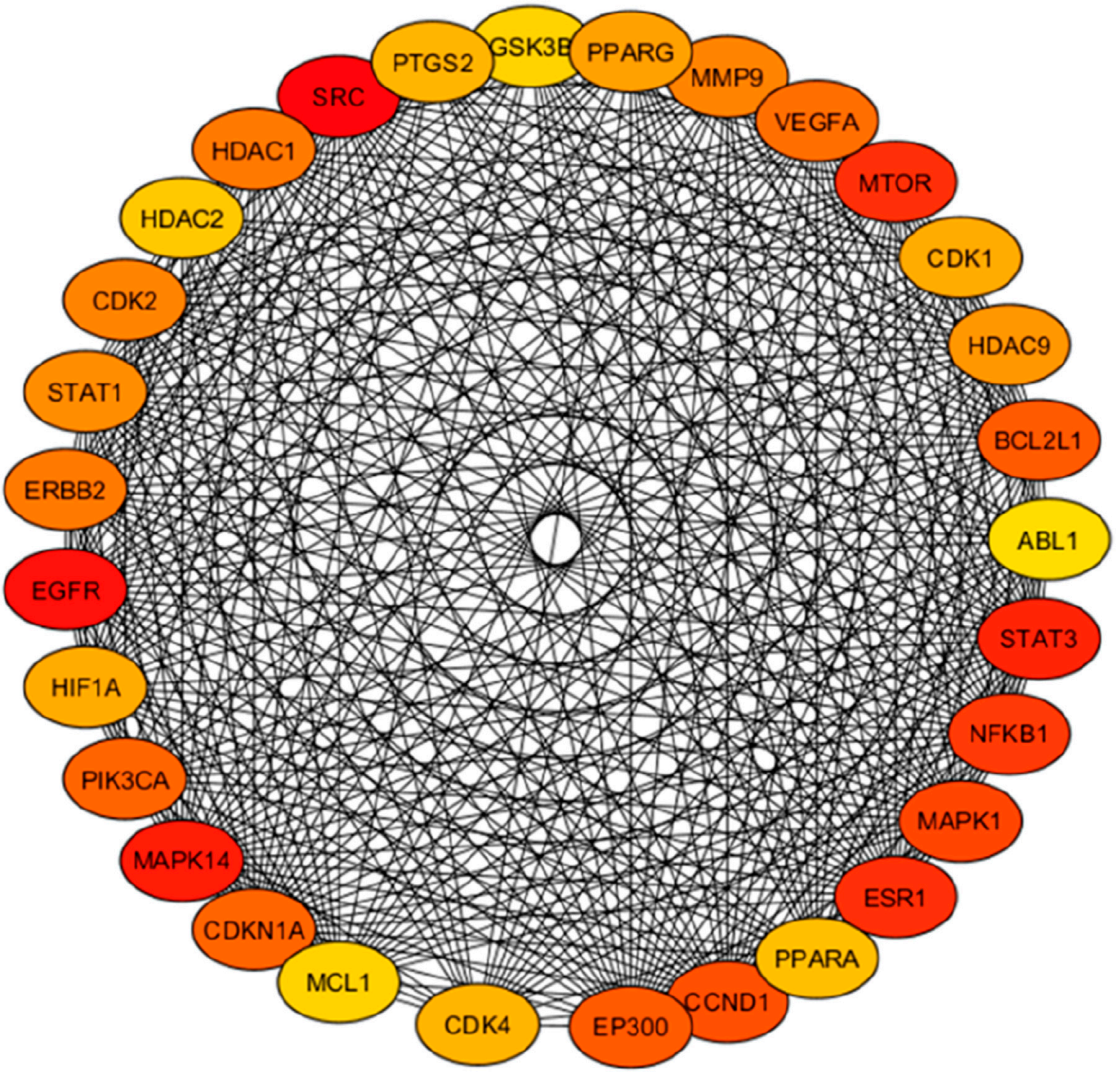
PPI network for the top hub targets. The top 30 targets selected from *Launaea cornuta* ethyl acetate-cervical cancer targets. The intensity of the colour represents the significance (*p* < 0.05) of the targets, with darker red indicating a higher degree.

The intersected targets obtained using the Venn tool were imported into the STRING database. Cytoscape 3.10 software was used to construct a PPI network, as shown in Figure 9B. The top 30 key hub targets were selected according to their degree and score ranking using the Cytohubba plugin: the greater the degree value, the larger the node (Supplementary Table S4). The PPI exhibited 124 nodes and 1,457 edges. The average node degree and Local Clustering Coefficient were 23.5 and 0.61, respectively. The expected number of edges was 720. The PPI enrichment *p-*value was <1.0e-16, implying there were more interactions of the proteins than expected. This could also indicate that proteins are biologically linked as cluster proteins. The top 30 hub genes are shown in Figure 10. Among the top 30 targets, AKT1, MDM2, and CDK2 were selected as therapeutic targets in cervical cancer due to their established roles in the regulation of apoptosis and BCL2, Caspase9, TP53 and P21 due to their established role in the regulation and progression of the cell cycle and apoptosis (Rashmi et al., 2014; Li et al., 2023; Tatekawa et al., 2024).

### Gene ontology enrichment analysis

**FIGURE 11:**
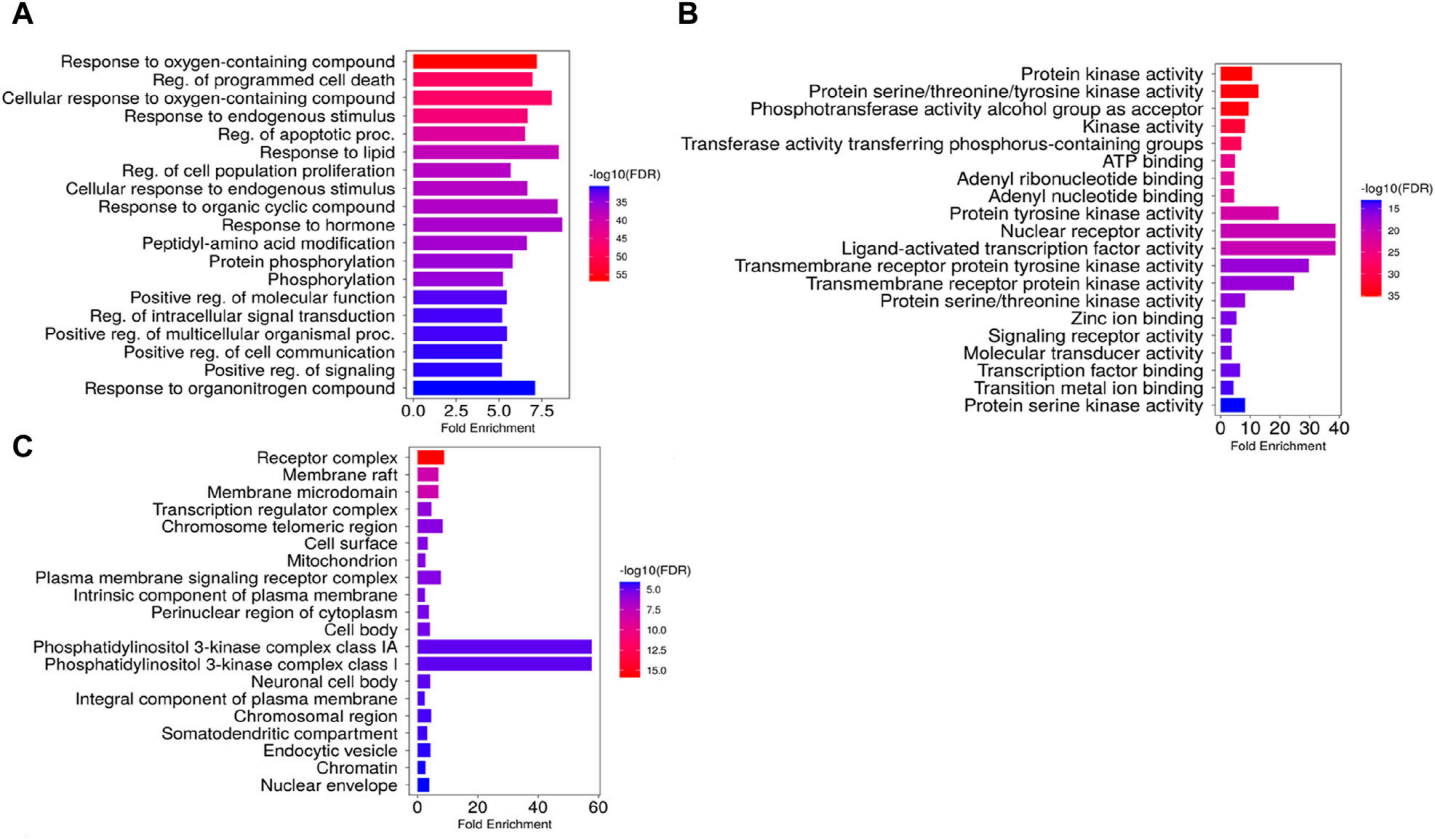
Gene Ontology enrichment terms associated with *Launaea cornuta* ethyl acetate compounds. **(A)** biological process terms, **(B)** molecular function terms, and **(C)** cellular component terms. The bar represents the GO terms on the vertical axis. The LogP values are shown on the horizontal axis. The enrichment colour scale defines the FDR, with red colour indicating a high FDR value and a significant association.

GO and KEGG enrichment analyses were conducted for the 124 intersection targets using ShinyGO 0.77 server (restricted species: *H. sapiens*; *p* < 0.05), and a total of 1415 GO enrichment terms were retrieved. The GO terms were classified into three categories with 1,234 terms in the biological processes (BP) category, 88 in the cellular components (CC) category and 93 in the molecular functions (MF) category. The top 20 significant GO terms for each GO category were selected (Figure 11). The top enriched biological processes mainly included MAP Kinase cascade, regulation of programmed cell death and apoptotic processes, response to both organic cyclic compounds and hormones, cellular response to oxygen-containing compounds, and positive regulation of cell population proliferation. The most enriched cellular compounds terms included phosphatidylinositol 3-kinase complexes I and IA, endosome, receptor complexes, transferase complex, and membrane raft, and the significantly enriched molecular function terms mainly associated with the core targets included nuclear receptors, ligand-activated transcription factors, transmembrane receptor protein tyrosine kinase, and protein tyrosine kinase activities.

### Kyoto encyclopaedia of genes and genomes pathway enrichment analysis

**FIGURE 12:**
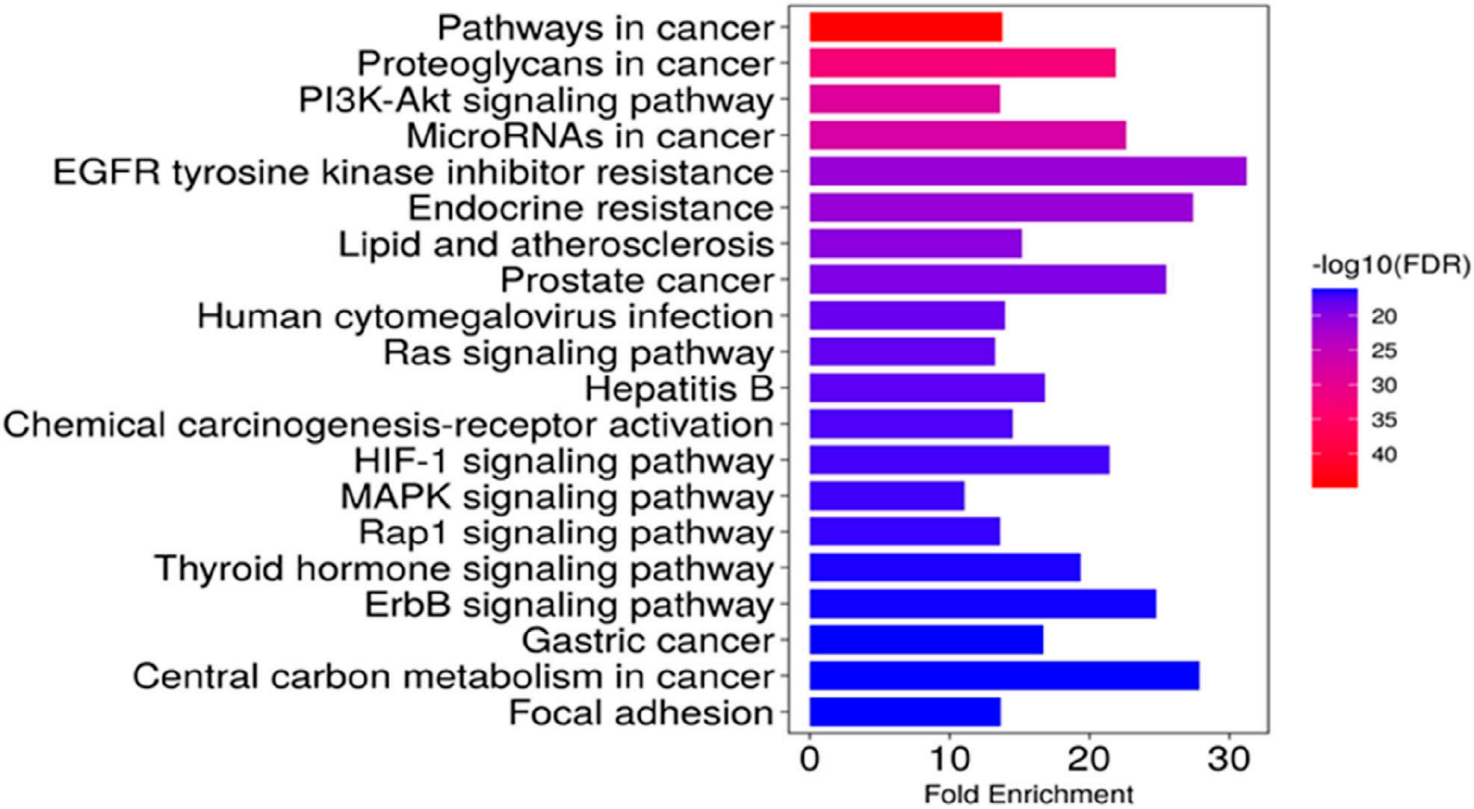
Kyoto Encyclopedia of Gene and Genomes (KEGG) pathway enrichment analysis. A) the enriched pathway associated with *Launaea cornuta* ethyl acetate targets. The bar represents the KEGG terms on the vertical axis. The LogP values are shown on the horizontal axis. The enrichment colour scale defines the FDR, with red colour indicating a high FDR value and a significant association.

**FIGURE 13:**
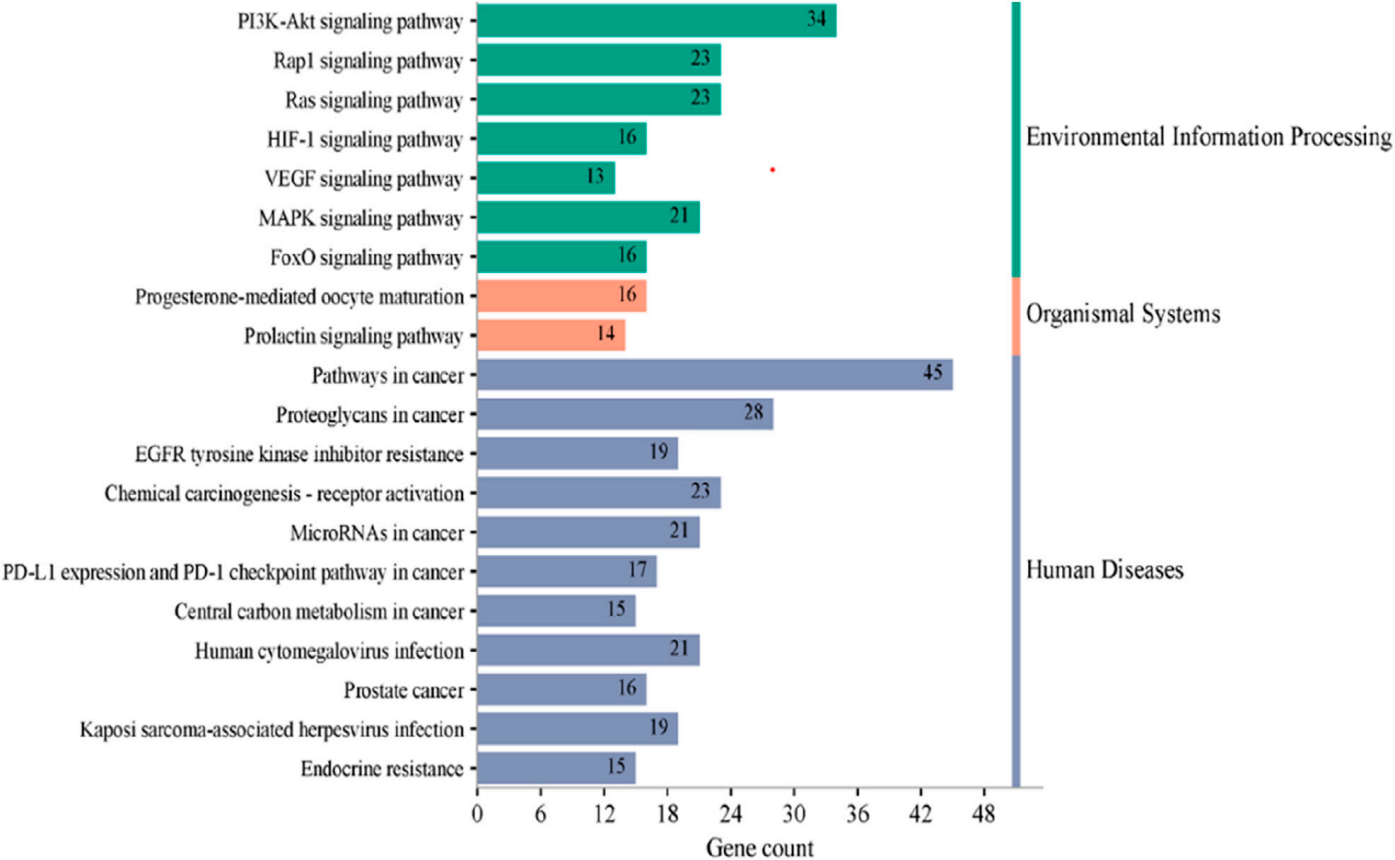
Enriched KEGG pathways associated with cervical cancer-related targets of *Launaea cornuta* ethyl acetate.

**FIGURE 14:**
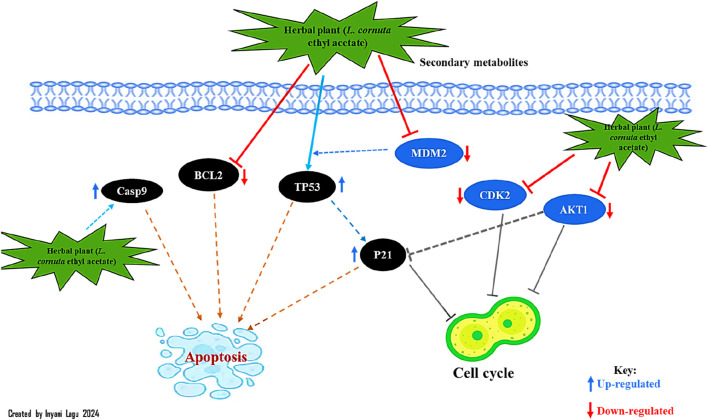
Proposed pathways and therapeutic modules of *Launaea cornuta* ethyl acetate fraction against cervical cancer.

KEGG pathway analysis was performed to explore the pathways associated with the anticervical cancer effect of the *L. cornuta* ethyl acetate-related target genes. The results showed that 124 target genes were associated with enriched in 123 significantly enriched pathways (*p >* 0.05). This implies that the anticervical cancer mechanism of *L. cornuta* ethyl acetate involves multiple targets, genes, and pathways. The top 20 KEGG signalling pathways with lower *p*-values were selected as the significant pathways (Figure 12; Supplementary Table S5). The top enriched cancer-associated pathways included central carbon metabolism in cancer, Ras signalling pathway, FoxO signalling pathway and PI3K-Akt signalling pathway. The PI3K-AKT signalling pathway with a lower *p*-value was predicted to be highly associated with cervical cancer and was selected for subsequent analysis. The KEGG pathway mapped to link molecular interaction, reactions, and relation network associated with top enriched pathway are presented in Figure 13. The proposed pathway model-based on the selected targets is presented in Figure 14.

### Molecular docking results

**FIGURE 15:**
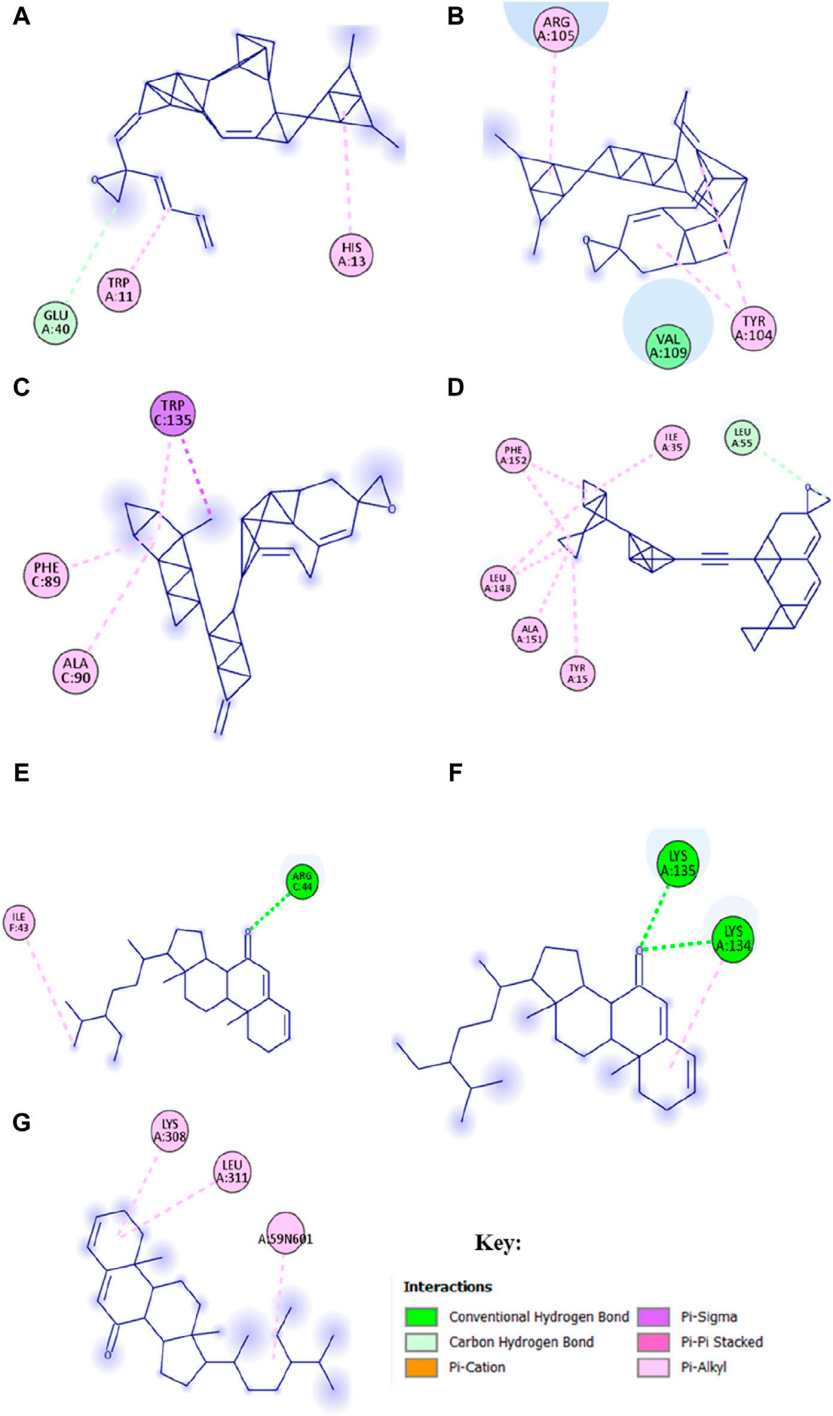
Molecular docking results showing 2D schematic representation of docking interactions of stigmasteryl methyl ether (M12) and tremulone (M7) with selected targets: **(A)** AKT1-M12; **(B)** MDM2-M12; **(C)** CDK2-M12; **(D)** BCL2-M12; **(E)** Casp9-M7; **(F)** TP53-M7 and **(G)** P21-M7.

**FIGURE 16:**
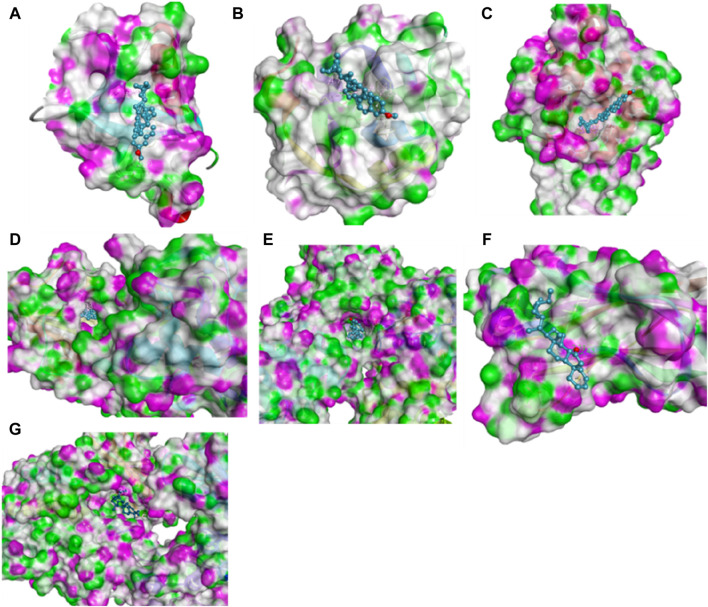
Molecular docking results showing 3D schematic presentation of M12 and M7 with target genes **(A)** AKT1-M12, **(B)** MDM2-M12, **(C)** CDK2-M12, **(D)** BCL2-M12, **(E)** Casp9-M7, **(F)** TP53-M7 and **(G)** P21-M7).

Molecular docking was performed to stimulate the possibility of binding and interactions between *L. cornuta* ethyl acetate fraction compounds and the core targets in cervical cancer using PyRx software. Six (6) compounds of *L. cornuta* ethyl acetate fraction were docked against 7 target proteins (Supplementary Table S6). We selected 3 target proteins (AKT1, CDK2, and MDM2 from the top core hub genes and 4 target proteins (BCL2, TP53, P21, and Casp9) due to their established roles in regulating cell cycle and apoptosis (Xing et al., 2015; Yuan et al., 2018; Ghosh et al., 2020; Luo et al., 2023). Two ligands, stigmasteryl methyl ether (M12) and tremulone (M7), that exhibited binding affinity less than −7.0 kcal/mol with zero (0) root mean standard deviation (RMSD), were selected, analysed and presented in Supplementary Table S7 and Supplementary Figure S3. Molecular docking results indicated that stigmasteryl methyl ether and tremulone ligands exhibited good binding affinity with the selected target proteins (CDK2-M12 with −12.6 kcal/mol, MDM2-M12 with −9.1 kcal/mol, BCL2-M12 with −10.3 kcal/mol, Casp9-M7 with −9.9 kcal/mol, P21-M7 with −8 kcal/mol, TP53-M7 with −7 kcal/mol and AKT-M12 with −9.5 kcal/mol respectively). Their interactions were mainly through several hydrogen and hydrophobic bonds (Figures 15 and 16).

The redocked results for the native ligands with their target proteins are presented in Supplementary Figure S4 and Supplementary Table S8. M12 bonds to AKT1 protein through one hydrogen interaction with residue Glu40A and two hydrophobic interactions with residues His13A and Trp11A. Meanwhile, the native ligand NL7 interacted with AKT1 protein (−5.2 kcal/mol) through several hydrogen bonds with residues Lys20A, Arg15A, and Glu17A and hydrophobic interactions with residues Glu85A, Glu17A, Lys20A and Arg15A. The interaction between M12 and MDM2 protein occurred through hydrophobic bonds only with interacting residues of Val109A, Tyr104A, and Arg105A, whereas the native ligand NL2 bind to MDM2 (−7.3 kcal/mol) through one hydrogen bond with residues Hu8201A and hydrophobic interactions with residues Hu8201A and Lys51A. M12 bind to CDK2 protein through one hydrogen interaction with residue Leu55A and hydrophobic interactions with residues Thr15A, Ala151A, Leu148A, Phe152A, and Ile35A. CDK2 interacted with its co-recrystallised ligand NL1 (−9.3 kcal/mol) through a number of hydrogen interactions with residues Thr14A, Gln131A, Glu12A, Asp145A, Asp127A, Phe146A, Lys33A and Leu148A as well as several hydrophobic interactions with residues Ala151A, Ile35A, Lys33A, Leu148A, Tyr15A, Asp127A and Asp145A respectively. BCL2 protein interacted with M12 through several hydrophobic interactions with residues Trp135C, Phe89C and Ala90C, while the native ligand NL3 binds to BCL2 (−8.1 kcal/mol) through two hydrogen bonds with residues Arg142A and His143A and hydrophobic bonds through residues Arg86A, Ala90A, Phe89A and Glu138A. The interaction between M7 and Casp9 occurred through residue Arg44C to form a hydrogen bond and residue Ile43 to form a hydrophobic bond. Conversely, the native ligand NL4 interacted with Casp9 protein (−9.3 kcal/mol) through several hydrogen bonds with residues Arg44C, Arg52C, Asn45C, Glu46C, Ser48E, and Pro47A only. M7 bind to TP53 protein through two hydrogen bonds with residues Tys134 and Tys135 and through residue Lys134 to form a hydrophobic interaction. The co-recrystallised ligand NL6 (−8 kcal/mol) interacted through residues Lys129A and Gly132A to form hydrogen bonds and through residues Met117A, Phe102A, Phe133A and Tyr123A to form hydrophobic bonds and lastly, the interaction between P21 and M7 occurred through residues Lys308A, Leu311A, A:59N601 forming hydrophobic bonds only whereas the native ligand NL5 (−8.5 kcal/mol) had interaction through residues Gly409A, Glu423A and Asp393A to form two hydrogen bonds and hydrophobic bonds with residues Leu311A, Phe410A and Ile312A. These results showed that CDK2-M12, BCL2-M12, Casp9-M7 and P21-M7 complexes shared the same active site residues implicated in the binding of the native ligand for each protein, whereas ATK1-M12, MDM2-M12 and TP53-M7 complexes did not share any active site residues implicated in the binding of native ligands.

### The effect of *L. cornuta* ethyl acetate fraction on mRNA expression levels of selected target genes in HeLa cells

**FIGURE 17:**
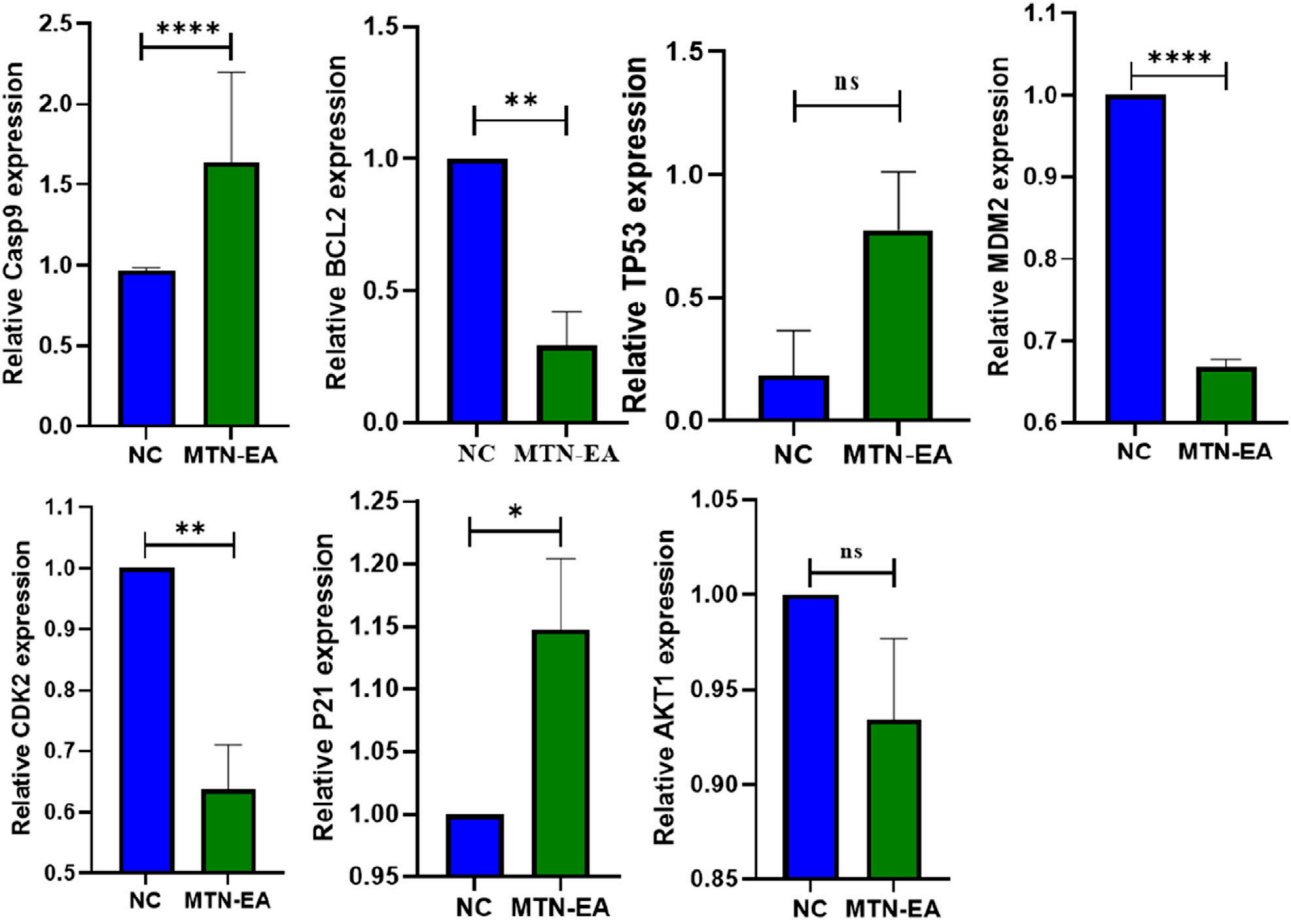
Relative gene expression analysis of *Launaea cornuta* ethyl acetate treated HeLa cells and the untreated control (0.2% DMSO). Mean ± SD values represent at least three independent experiments. The relative expression for each target was compared to their negative control (NC). ns, *p* > 0.05; *, *p* < 0.05; ** *p* < 0.01; *** *p* < 0.001; **** *p* < 0.0001.

Real-time PCR analysis was performed to validate the putative molecular targets of *L. cornuta* ethyl acetate fraction associated with the cervical cancer cells, as depicted by the network analysis results. We evaluated the gene expression levels of ATK1, BCL2, CDK2, MDM2, P21, TP53, and Casp9 in HeLa cells treated with *L. cornuta* ethyl acetate fraction and untreated HeLa cells. AKT1, CDK2 and MDM2 were among the top hub genes. We further selected BCL2, P21, TP53 and Casp9 genes due to their established role in apoptosis and cell cycle. The quantification cycle, also known as the threshold cycle (Ct), was determined, and relative gene expression levels of the target genes were normalized to GAPDH (Figure 17). There was significant upregulation of Casp9 (*p* < 0.0001) and P21 (*p* < 0.5) expression levels in HeLa cells treated with *L. cornuta* ethyl acetate fraction compared to the negative control (NC). There was a significant downregulation of BCL-2 (*p* < 0.01), MDM2 (*p* < 0.0001), and CDK2 (*p* < 0.01) expression levels in the *L. cornuta*-treated cervical cancer cells compared to the NC. The gene expression level of TP53 was upregulated, although the difference was not significant (*p* > 0.0591) in treated HeLa cells compared to the NC. Meanwhile, there was no significant expression level of AKT1 (*p* > 0.1452) in the *L. cornuta*-treated cervical cancer cells compared to the NC. The RT-qPCR results suggest the fact that *L. cornuta* ethyl acetate fraction interferes with cervical cancer cell proliferation by inducing cell cycle arrest and apoptosis.

## Discussion

Cervical cancer, among other cancers, remains one of the leading causes of mortality in women worldwide (Cohen et al., 2019; Kasi et al., 2021). Due to the increased global prevalence of cervical cancer and its poor prognosis, cervical cancer remains undetected in the early stages, resulting in patients with advanced stages; also, due to emerging drug resistance in cervical cancer treatment, there is a need for alternative drug design and development that involves target-specific therapies (Xi et al., 2023). Regardless of some chemotherapeutic drugs such as cisplatin, paclitaxel (Taxol), and carboplatin approved and recognised by the FDA of the United States, they tend to have adverse effects and resistance in patients with cervical cancer (Li et al., 2016). Furthermore, there are limited effective treatments for advanced cervical cancer. However, the high cost of cancer treatment is a significant constraint on patients’ access to quality healthcare in low-income countries. These populations tend to prefer traditional herbs that are relatively affordable and available. Research shows that natural products from medicinal herbs are increasingly valuable in developing anticancer drugs (Elshamy et al., 2019; Owolabi et al., 2020). In this regard, *L. cornuta* merits our attention because it has been widely used as a traditional medicine among Kenyan communities to treat various diseases, including cancers (Ibrahim, 2021; Chemweno et al., 2022).

Using MTT assay, we evaluated the antiproliferative activity of the crude extract, hexane, ethyl acetate and water fractions from aerial parts of *L. cornuta* against cervical cancer cell lines. Our results demonstrated selective antiproliferative effect of *L. cornuta* ethyl acetate fraction by inhibiting cellular proliferation in cervical cancer cells (HeLa-229) by less than 50% cell viability. The dose-dependent cytotoxicity test showed antiproliferative effects of *L. cornuta* ethyl acetate on HeLa cells in a dose-dependent manner with minimal cytotoxicity on non-cancerous cells. The ethyl acetate fraction of *L. cornuta* showed potent cytotoxic effects on HeLa cells as defined by the guidelines of the US National Cancer Institute (NCI), where an extract is generally considered to have *in vitro* cytotoxic activity if the IC_50_ value is ≤ 20 μg/mL after 48 h of incubation (Ogbole et al., 2017; Okpako et al., 2023b). We observed that the *L. cornuta* ethyl acetate fraction exhibited cytotoxicity on HeLa cells with an IC_50_ value of 20.56 μg/mL, thus presenting *L. cornuta* ethyl acetate fraction as a potential chemotherapeutic drug agent for cervical cancer. Previous studies reported the biological activity of *Launaea* species against various types of cancer cell lines, such as breast cancer, MCF-7, A549, and HCC cell lines (Rawat et al., 2016; Rezaei Seresht et al., 2016; Abouzied et al., 2021). Notably, this study is the first to report the anticancer activity of *L. cornuta* against HeLa cell line. On the other hand, *L. cornuta* ethyl acetate fraction did not display toxicity toward normal cells (CC_50_ of 48.83 μg/mL), setting a safety margin (selectivity index) of 2.38, which agreed with the safety margin threshold for drugs. A selectivity index greater than 2 is considered highly selective (Canga et al., 2022). Therefore, the anticancer activity exhibited by *L. cornuta* ethyl acetate fraction was not a result of general cellular toxicity compared with the doxorubicin, having a selective index of 1.64. Furthermore, *L. cornuta* ethyl acetate fraction demonstrated a substantial inhibitory effect on HeLa cell migration. It was noted that the wound size remained relatively open by almost 50% relative migration distance in HeLa cells treated with *L. cornuta* ethyl acetate fraction compared with the untreated cells. This result is consistent with findings from Khan *et al.* (2024) and Lin *et al.* (2022), where 6-gingerol and puerarin exhibited antimigration effects by suppressing the migration of PC-3 cancer cells and Ishikawa endometrial cancer cells, respectively (Lin et al., 2022; Khan et al., 2024). Thus, justifying the extract’s ability to prevent cancer cell metastases. Furthermore, we also studied the morphological changes in HeLa cells exposed to varying concentrations of the *L. cornuta* ethyl acetate fraction for 48 h. The data showed that *L. cornuta* ethyl acetate fraction triggered morphological changes in cells, including diminished HeLa cell viability with irregular and rounded shapes, detached from each other in a dose-dependent as compared with untreated HeLa cells, which remain in an organised monolayer with regular shapes. This finding tallied with the study reported by Ghosh *et al.* (2020) noted the atypical morphological changes of HeLa cells exposed to *Hexagonia glabra* after 24 h (Ghosh et al., 2020). In summary, these findings indicate the potential effects of *L. cornuta* ethyl acetate fraction in inhibiting proliferation in HeLa cells.

The preliminary screening of *L. cornuta* showed the presence of phenols, glycosides, tannins, and terpenoids in the ethyl acetate fraction of *L. cornuta*, which were also reported by previous findings in *L. cornuta* ethyl acetate aerial extract (Akimat et al., 2021; Maina et al., 2022). Thirteen (13) photochemical compounds were identified in *L. cornuta* ethyl acetate fraction through GC-MS analysis, and fatty acids and terpenoids were the most abundant compounds. These compounds were not similar to those reported by Machocho *et al.* (2014) in the aerial extract of *L. cornuta* ethyl acetate (Machocho et al., 2014). Some of the compounds identified in *L. cornuta* ethyl acetate fraction have been previously reported to exhibit anticancer (cytotoxic and antioxidant) activity against various cancer cell lines; these included ethyl (9Z,12Z,15Z)-octadeca-9,12,15-trienoate (fatty acids) (Chumkaew et al., 2014; Eid et al., 2021; Huang et al., 2022), Stigmast-5-ene, 3beta-methoxy- (phytosterols) (Chumkaew et al., 2014; Alvarez-sala et al., 2019; Jaiganesh et al.) and tremulone (terpenoids) (Gupta and Chaphalkar, 2016; Lehtonen and Kaarniranta, 2016). These compounds were reported to induce cytotoxic effects in cancer cells through at least one of the pathways: apoptosis, inhibition of cell cycle, invasion and metastasis (Shaikhaldein et al., 2022). However, some of the compounds identified in our study have not been reported to exhibit anticancer activity, and their presence in the ethyl acetate fraction of *L. cornuta* may suggest that they work synergistically with other compounds (Ibrahim et al., 2022). Given the presence of these compounds in *L. cornuta*, ethyl acetate fraction could be attributed to the cytotoxic effects of the plants on cervical cancer cells. However, further research should be done to evaluate the anticancer activity of these individual compounds identified in the ethyl acetate fraction of *L. cornuta*.

The Gene Ontology and Kyoto Encyclopaedia of Genes and Genome Analysis presented several pathways as well as other diseases and disorders for the top hub genes. GO enrichment analysis revealed the direct involvement of the identified compounds in the regulation or progression of cervical cancer through programmed cell death, cell population, apoptotic processes, positive regulation of molecular function, and mitogen-activated protein (MAP) signalling. The KEGG pathway analysis showed that PI3K-AKT1 signalling was a significantly enriched pathway for *L. cornuta* ethyl acetate compounds. The EGFR tyrosine kinase inhibitor pathway, the MAPK signalling pathway, the Ras signalling pathway, and the FoxO signalling pathway were also enriched, suggesting the use of *L. cornuta* ethyl acetate fraction in the development of multi-target drugs. The MAPK pathway is known to play a role in inducing apoptosis and cell cycle arrest in tumorigenic cells (Chen, 2020; Huang et al., 2020; Tatekawa et al., 2024). Adding to the evidence from the pathway enrichment for *L. cornuta* ethyl acetate, the plant extracts may be used in targeting prostate cancer, human cytomegalovirus infection, and endocrine resistance treatment. PI3K/Akt pathway is known to play a key role in cancer cell proliferation, metastasis, differentiation, and drug resistance (Xia et al., 2015; Arjumand et al., 2016). Previous studies have shown that activation of PI3K through Akt phosphorylation results in the regulation of cell proliferation, thus promoting tumour growth (Arjumand et al., 2016; Jiang et al., 2020; Ma et al., 2024). Potential drug-like compounds that can inhibit cancer cell progression by negatively inhibiting Akt phosphorylation and downregulation of Akt kinase, thus regulating the PI3K-Akt pathway, could be a potential anticancer drug (Tao et al., 2017; Mart et al., 2023). Therefore, targeting the PI3K/Akt signalling pathway and its downstream targets, such as STAT3 and mTOR, may be a therapeutic option for *L. cornuta* ethyl acetate.

To validate the results of the network analysis, molecular docking was performed. As shown by molecular docking results, *L. cornuta* ethyl acetate’ stigmasteryl methyl ether (M12) and tremulone (M7) exhibited good interaction toward AKT1, CDK2, MDM2, BCL2, TP53, P21, and Casp9 proteins with binding affinity ranging from −7.0 to −12.6 kcal/mol. A docking score of less than −5 kcal/mol has been shown to represent a good binding affinity (Zhao et al., 2022), which means that the lower the docking score, the more stable and stronger the affinity between compounds and targets. As shown by the docking scores, the binding energies of all docking were less than −7.0 kcal/mol, indicating that these target genes have good and stable binding affinity with stigmasteryl methyl ester and tremulone compounds, respectively. Moreover, the binding energy values of the compounds docked with the selected target proteins are lower than those of the native ligands redocked with the selected proteins. Therefore, these results implies that M7 and M12 compounds exhibited good binding affinity and can form more stable interactions with the targeted proteins in cervical cancer cells. M7 and M12 compounds bonds to the CDK2, BCL2, Casp9 and P21 proteins through the same active binding pocket implicated in the binding of the native co-recrystallised ligands, suggesting M7 and M12 compounds maybe orthosteric hits. Whereas, M7 and M12 interacted with AKT1, MDM2, and TP53 proteins through different hydrogen and hydrophobic interacting amino acid residues (active binding pocket), which are not implicated in the binding of the native co-recrystallised ligand of redocked, thus suggesting that M7 and M12 compounds could be an allosteric hit toward AKT1, MDM2 and TP53 proteins. The presence of hydrogen bonds, hydrophobic interactions, and van der Waals forces in the ligand-protein interactions are critical for protein-ligand stability. Hydrogen bonds improve specificity, hydrophobic interactions augment the drug effects, and van der Waals forces improve structural fit between molecules, resulting in increased drug efficacy and potency in therapeutic applications (Dhar and Roy, 2020; Nussinov et al., 2023).

The gene expression levels of P21 and Casp9 were found to be high in HeLa cells treated with the ethyl acetate fraction *of L. cornuta* (20.56 μg/mL) compared to the untreated control. Studies by Dalghi *et al.* (2023), and Zhou *et al.* (2024) reported that high expression levels of P21 in cancer cells are implicated in the promotion of cell cycle arrest and apoptosis (Dalghi et al., 2023; Zhou et al., 2024). BCL2 is a known inhibitor of apoptosis (Babakanrad et al., 2023; Branch et al., 2023; Hosseini et al., 2024). Cancer cells typically evade cell death by upregulating antiapoptotic proteins like BCL-2 or inhibiting pro-apoptotic proteins such as Casp9. BCL-2 inhibits mitochondrial apoptosis by binding to pro-apoptotic proteins and blocking pore formation and cytochrome c release. Caspases and upstream regulatory factors, such as p53, trigger apoptosis (Ghosh et al., 2020). As observed in our results, there was downregulation of BCL2 with concomitant upregulation of Casp9 in HeLa cells treated with *L. cornuta* ethyl acetate compared to the untreated HeLa cells; this pattern was also reported by Eltamany *et al.* (2022). MDM2 is a protein that directly regulates TP53 function and stability (Hou et al., 2019; Zhu et al., 2021); as noted in this study, MDM2 was significantly downregulated (*p* < 0.0001) and therefore, the expression level of TP53 was insignificantly expressed (*p* > 0.05) in treated HeLa cells compared to the untreated control. Study by Zhu *et al.* (2021) reported that downregulation of the expression level of MDM2 results in upregulation of TP53 expression, resulting in the induction of cell cycle arrest and apoptosis in cervical cancer cells (Zhu et al., 2021). CDK is well known to regulate the transition of the cell cycle from the G1 phase to the S phase, thus accelerating the S phase and the proliferation efficiency in cells. It is highly expressed in cancer cells (Liu et al., 2013; Tadesse et al., 2020; Bao et al., 2023). *L. cornuta* ethyl acetate fraction elicited a significant downregulation of CDK2 (*p* > 0.01), which is consistent with the study reported by Zhong *et al.* (2019) (Zhong et al., 2019). Therefore, the downregulation of CDK2 in HeLa cells could be attributed to the anticancer effect of the *L. cornuta* ethyl acetate fraction through suppression of the cell cycle. Furthermore, the expression level of AKT1 was observed to be insignificant in treated HeLa cells despite its crucial role in the regulation of cell survival, angiogenesis, and tumorigenesis of cancer (Rashmi et al., 2014).

## Conclusion

The *L. cornuta* ethyl acetate fraction demonstrates significant antiproliferative effects against cervical cancer HeLa cells, with minimal cytotoxicity on non-cancerous cells and rich composition of fatty acids and terpenoids identified through GC-MS analysis. Network analysis has pinpointed drug-like compounds, including 2-linoleolglycerol, tremulone, spiro [2.5] non-4-ene, 4-ethyl-8-methylene-5-trimethylsilyl, ethyl (9Z, 12Z, 15Z)-octadeca-9,12,15-trienoate, stigmasteryl methyl ether and tricylo [20.8.0.0 (7,16)] triacontane, 1(22),7(16)-diepoxy targeting key proteins involved in cancer progressions and tumorigenesis, such as AKT1, MDM STAT3, EGFR, CDK2, MTOR, MAPK3 PTGS2, MCL1 and TNF among others. Intriguingly, the gene expression levels of BCL2, CDK2, MDM2, Casp9 and P21 were reversed in HeLa cells treated with *L. cornuta* ethyl acetate fraction when compared with the untreated HeLa cells, indicating its potential to regulate cell proliferation, apoptosis, and cell cycle in cancer cells. These findings highlight *L. cornuta’s* promise as a multi-target anticancer drug, warranting further *in-vitro* and *in vivo* studies to assess its efficacy and safety for clinical use as well as anticancer activity on other cancer cell lines.

## Data Availability

The datasets presented in this study can be found in online repositories. The names of the repository/repositories and accession number(s) can be found in the article/Supplementary Material.
