# Supplementary material for: Phytochemical analysis, in-vitro and in-silico study of antiproliferative activity of ethyl acetate fraction of Launaea cornuta (Hochst. ex Oliv. & Hiern) C. Jeffrey against human cervical cancer cell line
Source: Front Pharmacol. 2024 Jun 28;15:1399885. doi: 10.3389/fphar.2024.1399885 (PMC11239972; doi:10.3389/fphar.2024.1399885)
Supplement: Supplementary file 1 [file DataSheet1.docx]

**Phytochemical Analysis, In-Vitro and In-Silico Study of Antiproliferative Activity of Ethyl Acetate Fraction of *Launaea cornuta*** **(Hochst. ex Oliv. & Hiern) C. Jeffrey** **against Human Cervical Cancer Cell Line**

**Inyani John Lino Lagu^1*^, Dorothy Wavinya Nyamai ^2^, Sospeter Ngoci Njeru^3*^**

**^1^** Department of Molecular Biology and Biotechnology, Pan African University Institute for Basic Sciences, Technology and Innovation, Nairobi, Kenya; [lagu.inyani@students.jkuat.ac.ke](mailto:lagu.inyani@students.jkuat.ac.ke/)

**^2^** Department of Biochemistry, School of Biomedical Sciences, College of Health Sciences, Jomo Kenyatta University of Agriculture and Technology, Nairobi, Kenya; [dnyamai@jkuat.ac.ke](mailto:dnyamai@jkuat.ac.ke)

**^3^** Centre for Traditional Medicine and Drug Research (CTMDR), Kenya Medical Institute (KEMRI), Nairobi, Kenyan; [snjeru@kemri.go.ke](mailto:snjeru@kemri.go.ke)

**^*^** Correspondence: [snjeru@kemri.go.ke](mailto:snjeru@kemri.go.ke) (S.N.N.); [lagu.inyani@students.jkuat.ac.ke](mailto:lagu.inyani@students.jkuat.ac.ke) (I.J.L.L)

**Supplementary Results**

1. ****

**(B)**

**Supplementary Figure S1**. Cell viability assay at different concentrations of doxorubicin against A) HeLa cells and B) Vero cells after 48 h of incubation.

**Supplementary Table S1.** Primer sequences designed for qPCR

| Gene | Primer sequence |
| --- | --- |
| MDM2 | F: 5'-GATGGTGAGGAGCAGGCA-3'  R: 5'-TGCCTGCTCCTCACCATC-3' |
| CDK2 | F: 5'-TCTTTGCTGAGATGGTGACTCG-3'  R: 5'-TGTTAGGGTCGTAGTGCAGC-3' |
| AKT2 | F: 5'-TCCTTGCTTTCAGGGCTGCT-3'  R: 5'-GCCACACGATACCGGCAAAG-3' |
| BCL2 | F: 5'-GGCCTCAGGGAACAGAATGAT-3'  R: 5'-TCCTGTTGCTTTCGTTTCTTTC-3' |
| Caspase9 | F: 5'-CCTGCCCGCTGTTTGGA-3'  R: 5'-GCTGGGAAATGGGGAGACAA-3' |
| TP53 | F: 5'-CTTCGAGATGTTCCGAGAGC-3'  R: 5'-GACCATGAAGGCAGGATGAG-3' |
| P21 | F: 5'-GCGACTGTGAGCTAATG-3'  R: 5'-TTAGAAGCTTGGCAAAGGGC-3' |
| GAPDH | F: 5'-GTGGCTGGCTCAAAAAGG-3'  R: 5'-GGGGAGATTCAGTGTGGTGG-3' |

Key: F represent forward primer; R represents reverse primer

**Supplementary Table S2. Phytochemical classes of *L. cornuta* extract and the fractions**

| Phytochemicals | MTN-Crude | MTN-ethyl acetate |
| --- | --- | --- |
| Alkaloids | - | - |
| Glycosides | ++ | +++ |
| Phenols | +++ | ++ |
| Flavonoids | - | - |
| Steroids | - | - |
| Saponins | ++ | - |
| Terpenoids | +++ | ++ |
| Quinones | + | ++ |
| Tannins | ++ | - |

Key: - (absent), + (low amount), ++ (moderate amount), and +++ (high amount).


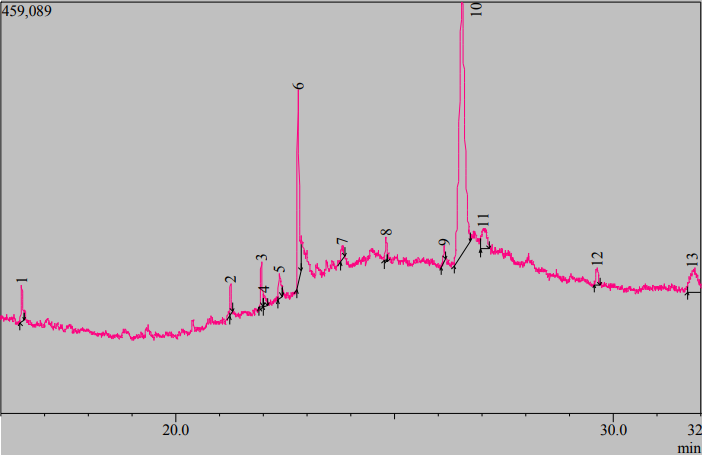


**Supplementary Figure S2**. A chromatogram obtained from a GC-MS analysis of the *L. cornuta* ethyl acetate fraction

**Supplementary Table** **S3. Prediction of the physiochemical properties for *L. cornuta* ethyl acetate** **compounds based on the Lipinski (mainly considered here), Veber, Egan, Muegge, and Ghose rules.**

| Ligands  Number | Physical and chemical properties | | | | | | Lipinski  Violations | Veber Violations | Egan Violations | Ghose Violations | Muegge Violations | BBB |
| --- | --- | --- | --- | --- | --- | --- | --- | --- | --- | --- | --- | --- |
|  | MW (g/mol) | Molar refractive index | Rotatable  bonds number | LogP (Octanol/Water) | H-bond  Acceptors Number | H- bonds donors Number | Categorical  (Yes/No) | | | | | |
| Threshold | ≤500 | 40≤MR≤130 | ≤10 | ≤5 | ≤10 | ≤5 | Yes/No | Yes/No | Yes/No | Yes/No | Yes/No | Yes/No |
| M1 | 196.2 | 52.51 | 0 | 1.49 | 3 | 1 | Yes;1 | Yes | Yes | Yes | No | Yes |
| M2 | 568.9 | 174.09 | 34 | 6.27 | 5 | 1 | No;2 | No | No | No | No | Yes |
| M3 | 236.4 | 68.95 | 2 | 2.74 | 2 | 2 | Yes;1 | Yes | Yes | Yes | Yes | Yes |
| M4 | 505.8 | 113.85 | 6 | 5.26 | 10 | 0 | No;2 | Yes | No | No | No | Yes |
| M5* | 354.5 | 105.72 | 18 | 3.42 | 4 | 2 | Yes;1 | No | Yes | Yes | No | **No** |
| M6 | 266.5 | 87.21 | 14 | 4.4 | 1 | 1 | Yes;1 | No | Yes | No | No | Yes |
| M7* | 410.7 | 131.79 | 6 | 6.53 | 1 | 0 | Yes;1 | Yes | No | No | No | **No** |
| M8* | 234.5 | 76.65 | 2 | 4.63 | 0 | 0 | Yes;1 | Yes | Yes | Yes | No | **No** |
| M9 | 185.0 | 46.41 | 1 | 2.9 | 0 | 0 | Yes;1 | Yes | Yes | Yes | No | Yes |
| M10 | 298.9 | 92.69 | 14 | 4.82 | 1 | 0 | Yes;1 | No | No | No | No | **No** |
| M11* | 306.5 | 98.12 | 15 | 4.84 | 2 | 0 | Yes;1 | No | No | No | No | **No** |
| M12* | 428.7 | 137.96 | 7 | 6.92 | 1 | 0 | Yes;1 | Yes | No | No | No | **No** |
| M13* | 444.7 | 138.08 | 0 | 6.14 | 2 | 0 | Yes;1 | Yes | No | No | No | **No** |

***Key****: MW, molecular weight, BBB, brain-blood barrier, *, drug-like compounds (DL). M1 to M2 represent peaks 1 to 13 from Table 1.*

**Supplementary Table S4**. The topological properties of the 122 target proteins from PPI for *L. cornuta* ethyl acetate-cervical cancer. The top 30 key hub targets were extracted from this table based on the degree and score ranking indicated.

| **Targets** | **Degree** | **Rank** | | **BC (Betweenness centrality)** | **CC (Closeness Centrality)** | **Neighbourhood**  **Connectivity** |
| --- | --- | --- | --- | --- | --- | --- |
| EGFR | 79 | 1 | | 0.055208 | 0.724551 | 32.11392 |
| AKT1* | 77 | 2 | | 0.071777 | 0.720238 | 31.63636 |
| STAT3 | 77 | 3 | | 0.095275 | 0.724551 | 32.33766 |
| TNF | 75 | 4 | | 0.064239 | 0.711765 | 31.14667 |
| HIF1A | 64 | 5 | | 0.031084 | 0.664835 | 34.9375 |
| ESR1 | 64 | 6 | | 0.054245 | 0.664835 | 33.70313 |
| PPARG | 59 | 7 | | 0.045194 | 0.643617 | 33.76271 |
| MAPK3 | 59 | 8 | | 0.034409 | 0.647059 | 33.9322 |
| PTGS2 | 57 | 9 | | 0.055984 | 0.643617 | 34.01754 |
| PIK3CA | 54 | 10 | | 0.015973 | 0.617347 | 36.40741 |
| MTOR | 53 | 11 | | 0.010598 | 0.620513 | 39.03774 |
| MDM2* | 51 | 12 | | 0.024793 | 0.611111 | 36.19608 |
| MAPK1 | 49 | 13 | | 0.019053 | 0.605 | 36.61224 |
| TLR4 | 48 | 14 | | 0.016853 | 0.605 | 36.04167 |
| GSK3B | 48 | 15 | | 0.013596 | 0.60804 | 37.72917 |
| KDR | 48 | 16 | | 0.011173 | 0.60804 | 38.33333 |
| PARP1 | 47 | 17 | | 0.013502 | 0.605 | 37.68085 |
| JAK2 | 46 | 18 | | 0.021973 | 0.590244 | 38.54348 |
| KIT | 46 | 19 | | 0.005944 | 0.59901 | 40.02174 |
| ABL1 | 45 | 20 | | 0.026844 | 0.581731 | 35 |
| FGF2 | 44 | 21 | | 0.008222 | 0.587379 | 38.86364 |
| MCL1 | 42 | 22 | | 0.008108 | 0.584541 | 39.7381 |
| PGR | 40 | 23 | | 0.007748 | 0.578947 | 40 |
| EZH2 | 39 | 24 | | 0.005949 | 0.57346 | 39.97436 |
| AR | 38 | 25 | | 0.014009 | 0.57346 | 39.71053 |
| MAPK14 | 38 | 26 | | 0.005725 | 0.568075 | 41.92105 |
| CDK2* | 36 | 27 | | 0.004725 | 0.565421 | 41.66667 |
| IL2 | 36 | 28 | | 0.002669 | 0.565421 | 43.38889 |
| IGF1R | 36 | 29 | | 0.002637 | 0.57346 | 44.08333 |
| MAPK8 | 35 | | 30 | 0.004545 | 0.565421 | 43.08571 |
| PIK3CB | 34 | | 31 | 0.00243 | 0.545045 | 39.5 |
| PRKCA | 31 | | 32 | 0.005817 | 0.55 | 40.41935 |
| MET | 31 | | 33 | 0.001846 | 0.555046 | 44.41935 |
| TERT | 30 | | 34 | 0.001658 | 0.547511 | 46.23333 |
| SYK | 30 | | 35 | 0.005843 | 0.547511 | 39.76667 |
| PPARA | 29 | | 36 | 0.019876 | 0.55 | 36.75862 |
| NTRK1 | 29 | | 37 | 0.00695 | 0.542601 | 42.75862 |
| PIK3CD | 29 | | 38 | 0.001303 | 0.526087 | 41.31034 |
| TLR2 | 28 | | 39 | 0.006484 | 0.540179 | 40.17857 |
| FLT1 | 28 | | 40 | 0.002244 | 0.542601 | 42.92857 |

| RAF1 | 27 | 41 | 0.002461 | 0.530702 | 42.92593 |
| --- | --- | --- | --- | --- | --- |
| CDK1 | 27 | 42 | 0.002202 | 0.528384 | 41.59259 |
| CSF1R | 27 | 43 | 0.00177 | 0.537778 | 42.85185 |
| PIK3CG | 26 | 44 | 0.007638 | 0.53304 | 45.23077 |
| CTSB | 26 | 45 | 0.010178 | 0.535398 | 37.26923 |
| CCR2 | 26 | 46 | 0.003239 | 0.53304 | 39.84615 |
| NR3C1 | 26 | 47 | 0.002974 | 0.542601 | 44.07692 |
| BRD4 | 26 | 48 | 0.003209 | 0.526087 | 38.42308 |
| ESR2 | 25 | 49 | 0.004211 | 0.537778 | 45.8 |
| AURKA | 25 | 50 | 0.00138 | 0.52381 | 44.2 |
| FGFR2 | 24 | 51 | 9.74E-04 | 0.514894 | 41.75 |
| PLAU | 24 | 52 | 0.00253 | 0.53304 | 41.70833 |
| PTPN6 | 24 | 53 | 0.001282 | 0.510549 | 40.70833 |
| ALK | 24 | 54 | 0.001218 | 0.52381 | 43.25 |
| ABCG2 | 24 | 55 | 0.018682 | 0.530702 | 43.20833 |
| JAK3 | 22 | 56 | 8.69E-04 | 0.508403 | 42.04545 |
| PRKCD | 22 | 57 | 0.001615 | 0.517094 | 42.68182 |
| PKM | 22 | 58 | 0.010168 | 0.530702 | 43.63636 |
| CYP19A1 | 21 | 59 | 0.010026 | 0.530702 | 43.66667 |
| PTGS1 | 21 | 60 | 0.006779 | 0.517094 | 35.2381 |
| TOP2A | 20 | 61 | 0.001653 | 0.502075 | 36.85 |
| MMP7 | 20 | 62 | 0.001225 | 0.517094 | 43.85 |
| TGFBR1 | 20 | 63 | 5.87E-04 | 0.519313 | 47.65 |
| IDO1 | 19 | 64 | 3.85E-04 | 0.514894 | 47.21053 |
| MMP1 | 19 | 65 | 3.88E-04 | 0.514894 | 46.78947 |
| APOB | 19 | 66 | 0.007461 | 0.512712 | 37.05263 |
| RARA | 19 | 67 | 0.002666 | 0.510549 | 43.10526 |
| PLK1 | 17 | 68 | 8.36E-04 | 0.476378 | 35.05882 |
| ROCK1 | 17 | 69 | 0.0013 | 0.49187 | 42.88235 |
| VCP | 17 | 70 | 0.001744 | 0.495902 | 37.47059 |
| CDC25A | 17 | 71 | 8.88E-04 | 0.482072 | 37.47059 |
| CTSS | 16 | 72 | 8.36E-04 | 0.5 | 37.4375 |
| CD38 | 16 | 73 | 4.64E-04 | 0.49187 | 42.875 |
| KAT5 | 15 | 74 | 4.52E-04 | 0.493878 | 44.66667 |
| AURKB | 14 | 75 | 4.39E-04 | 0.436823 | 29.5 |
| PTGER4 | 14 | 76 | 0.003492 | 0.5 | 40.71429 |
| ROCK2 | 13 | 77 | 8.67E-04 | 0.493878 | 44.76923 |
| TRPV1 | 13 | 78 | 0.016844 | 0.49187 | 44.23077 |
| TEK | 13 | 79 | 1.80E-04 | 0.487903 | 52.15385 |
| COL18A1 | 13 | 80 | 3.83E-04 | 0.476378 | 40.30769 |
| MDM4 | 13 | 81 | 9.82E-05 | 0.47451 | 45.38462 |
| SPHK1 | 12 | 82 | 0.016687 | 0.495902 | 46.83333 |
| TRIM24 | 11 | 83 | 1.82E-04 | 0.478261 | 43.54545 |
| PTGES | 11 | 84 | 0.001263 | 0.468992 | 34.18182 |
| APEX1 | 11 | 85 | 2.67E-04 | 0.480159 | 45.27273 |
| PTGER3 | 10 | 86 | 7.45E-04 | 0.453184 | 30.6 |
| GRIN2B | 10 | 87 | 0.003398 | 0.467181 | 40 |
| CTSL | 10 | 88 | 3.91E-04 | 0.461832 | 35.5 |
| AKR1C3 | 10 | 89 | 0.017398 | 0.456604 | 24.5 |
| NR1I2 | 10 | 90 | 0.005612 | 0.467181 | 29.3 |
| HPGD | 10 | 91 | 0.003183 | 0.438406 | 21.5 |
| CYP27B1 | 9 | 92 | 4.46E-04 | 0.487903 | 42 |
| FABP4 | 9 | 93 | 9.55E-04 | 0.468992 | 37 |
| EPHB2 | 9 | 94 | 0.002035 | 0.449814 | 28.77778 |
| SHBG | 8 | 95 | 6.67E-05 | 0.467181 | 42.625 |
| SLC9A1 | 8 | 96 | 7.97E-04 | 0.456604 | 41 |
| PTGER2 | 8 | 97 | 2.35E-04 | 0.408784 | 20.125 |
| EPHA3 | 8 | 98 | 0.001781 | 0.448148 | 34 |
| P2RX7 | 7 | 99 | 1.53E-04 | 0.443223 | 34 |
| CDK9 | 7 | 100 | 5.51E-06 | 0.456604 | 49.28571 |
| BCHE | 6 | 101 | 6.75E-05 | 0.470817 | 49.16667 |
| EPHA1 | 6 | 102 | 8.34E-04 | 0.438406 | 28.66667 |
| DUSP3 | 5 | 103 | 0 | 0.44 | 52 |
| FABP5 | 5 | 104 | 2.00E-05 | 0.448148 | 38.6 |
| LPAR3 | 4 | 105 | 1.65E-04 | 0.424561 | 36.25 |
| SOAT1 | 4 | 106 | 7.41E-04 | 0.387821 | 17.25 |
| AHR | 4 | 107 | 1.84E-04 | 0.411565 | 34.25 |
| ACACB | 4 | 108 | 7.24E-05 | 0.417241 | 29.75 |
| EPHB6 | 4 | 109 | 0 | 0.375776 | 17 |
| SLC10A2 | 4 | 110 | 2.99E-04 | 0.380503 | 16.75 |
| ABCC4 | 4 | 111 | 5.52E-04 | 0.371166 | 12 |
| NDUFA13 | 3 | 112 | 0.032782 | 0.427562 | 27 |
| LPAR2 | 3 | 113 | 0 | 0.39934 | 29 |
| NR1H2 | 3 | 114 | 0 | 0.433692 | 50.66667 |
| DHCR24 | 3 | 115 | 4.62E-04 | 0.387821 | 25.66667 |
| CA12 | 3 | 116 | 8.60E-05 | 0.424561 | 45.33333 |
| NDUFB8 | 2 | 117 | 0 | 0.300995 | 2.5 |
| NDUFS6 | 2 | 118 | 0 | 0.300995 | 2.5 |
| CHRNA4 | 2 | 119 | 2.51E-05 | 0.382911 | 28 |
| UGCG | 1 | 120 | 0 | 0.332418 | 12 |
| KCNK3 | 1 | 121 | 0 | 0.330601 | 13 |
| AKR1C1 | 1 | 122 | 0 | 0.314286 | 10 |

**Supplementary Table S5.** Top 20 enriched KEGG pathways with 124 key targets of *L. cornuta* ethyl acetate in cervical cancer

| Fold Enrichment | Pathway Name | *P-*value | Gene Count |
| --- | --- | --- | --- |
| 15.10 | Pathways in cancer | 7.92E-40 | 45 |
| 18.09 | PI3K-Akt signalling pathway | 2.88E-31 | 34 |
| 26.11 | Proteoglycans in cancer | 4.33E-30 | 28 |
| 45.31 | EGFR tyrosine kinase inhibitor resistance | 5.37E-25 | 19 |
| 21.10 | Chemical carcinogenesis | 6.97E-23 | 23 |
| 20.63 | Rap1 signalling pathway | 2.61E-22 | 23 |
| 24.57 | MicroRNAs in cancer | 5.45E-22 | 21 |
| 18.76 | Ras signalling pathway | 1.80E-21 | 23 |
| 35.99 | PD-L1 expression and PD-1 checkpoint pathway in cancer | 9.37E-21 | 17 |
| 40.37 | Central carbon metabolism in cancer | 3.69E-19 | 15 |
| 17.66 | Human cytomegalovirus infection | 3.72E-19 | 21 |
| 31.08 | Prostate cancer | 1.41E-18 | 16 |
| 30.14 | Progesterone-mediated oocyte maturation | 2.20E-18 | 16 |
| 27.65 | HIF-1 signalling pathway | 8.75E-18 | 16 |
| 18.45 | Kaposi sarcoma-associated herpesvirus infection | 9.02E-18 | 19 |
| 37.68 | Prolactin signalling pathway | 1.34E-17 | 14 |
| 29.75 | Endocrine resistance | 3.06E-17 | 15 |
| 41.51 | VEGF signalling pathway | 5.53E-17 | 13 |
| 13.46 | MAPK signalling pathway | 6.35E-17 | 21 |
| 23.01 | FoxO signalling pathway | 1.32E-16 | 16 |

**Supplementary Table S6**. Molecular docking results display the negative free binding energy score for the docked ligands and targeted genes.

| Compound/Target | ID | CDK2 | MDM2 | BCL2 | CASP9 | P21 | TP53 | | AKT1 |
| --- | --- | --- | --- | --- | --- | --- | --- | --- | --- |
| (9Z,12Z)-octadeca-9,12-dienoyl chloride | M10 | -5.4 | -4.3 | -5.3 | -5.2 | -5.7 | | -4.1 | -4.1 |
| 2-Linoleoylglycerol | M5 | -6.5 | -4.5 | -5.7 | -4.1 | -6.2 | -4.3 | | -4.8 |
| Ethyl (9Z,12Z,15Z)-octadeca-9,12,15-trienoate | M11 | -5 | -4.8 | -4.8 | -4.4 | 5.9 | -4.4 | | -4.3 |
| Stigmast-5-ene, 3beta-methoxy- | M12 | -12.6 | -9.1 | -10.3 | -9.9 | -10 | -9 | | -9.5 |
| Tremulone | M7 | -8.8 | -6.4 | -7.2 | -7.5 | -8 | -7 | | -7.2 |
| Tricyclo [20.8.0.0(7,16)] triacontane, 1(22),7(16)-diepoxy | M13 | -8.4 | -6.9 | -8.8 | -9.8 | -10.1 | -6.7 | | -6.9 |

**Supplementary Table S7.** Binding affinities and receptor-ligand interactions of *L. cornuta* ethyl acetate fraction compounds with selected hub target genes

| Protein-Ligand complex | Binding affinity (kcal/mol) | Interaction residues | Interaction bond type |
| --- | --- | --- | --- |
| AKT1-M12 | -9.5 | His13A, Trp11A, Glu40A | Hydrogen and hydrophobic bonds |
| MDM2-M12 | -9.1 | Arg105A, Tyr104A | Hydrophobic bond |
| CDK2-M12 | -12.6 | Thr15A, Ala51A, Leu148A, Phe152A, Ile35A, Leu55A | Hydrogen and hydrophobic bonds |
| BCL2-M12 | -10.3 | Trp135C, Phe89C, Ala90A | Hydrophobic bond |
| Casp9-M7 | -9.9 | Arg44C, Ile43F | Hydrogen and hydrophobic bonds |


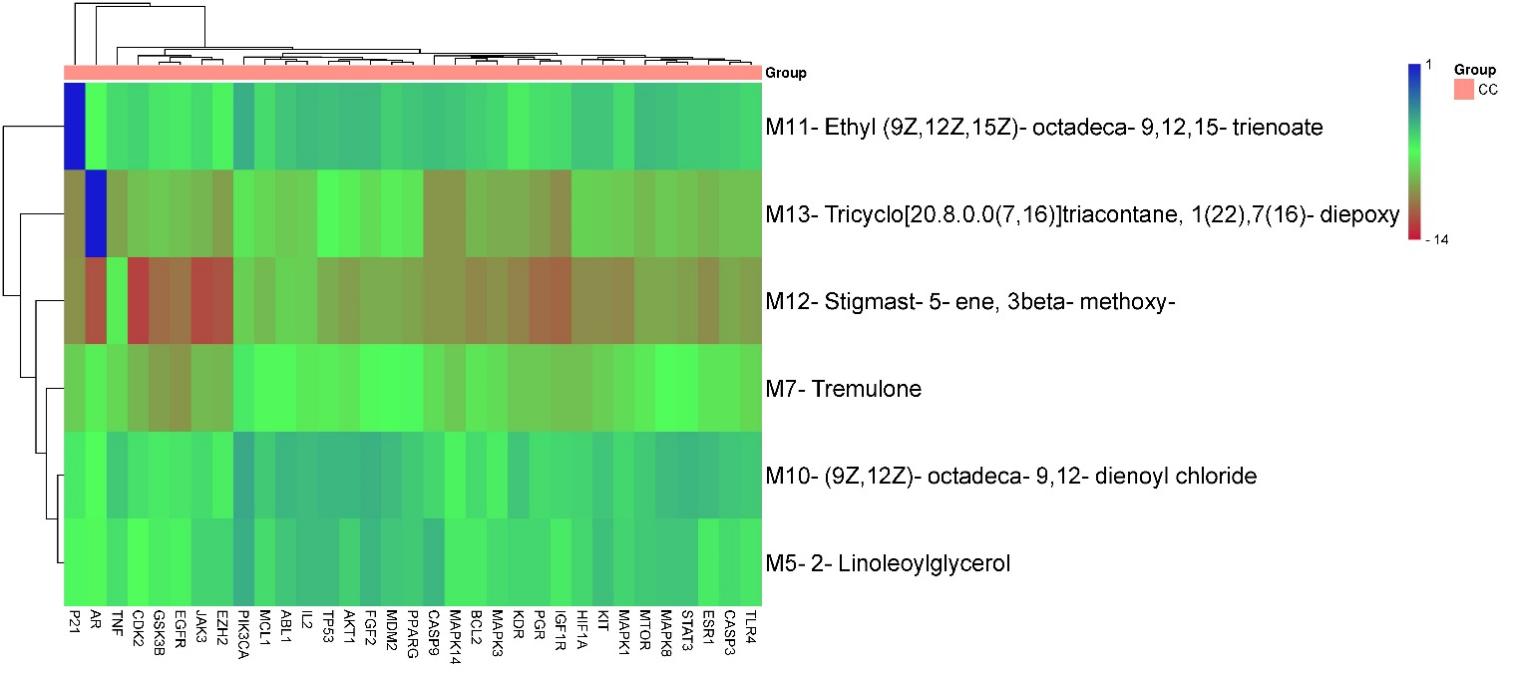


**Supplementary Figure S3**. Heatmap for the distribution of binding affinities obtained after molecular docking of 6 MTN-EA phytochemicals with 30 top hub genes in cervical cancer


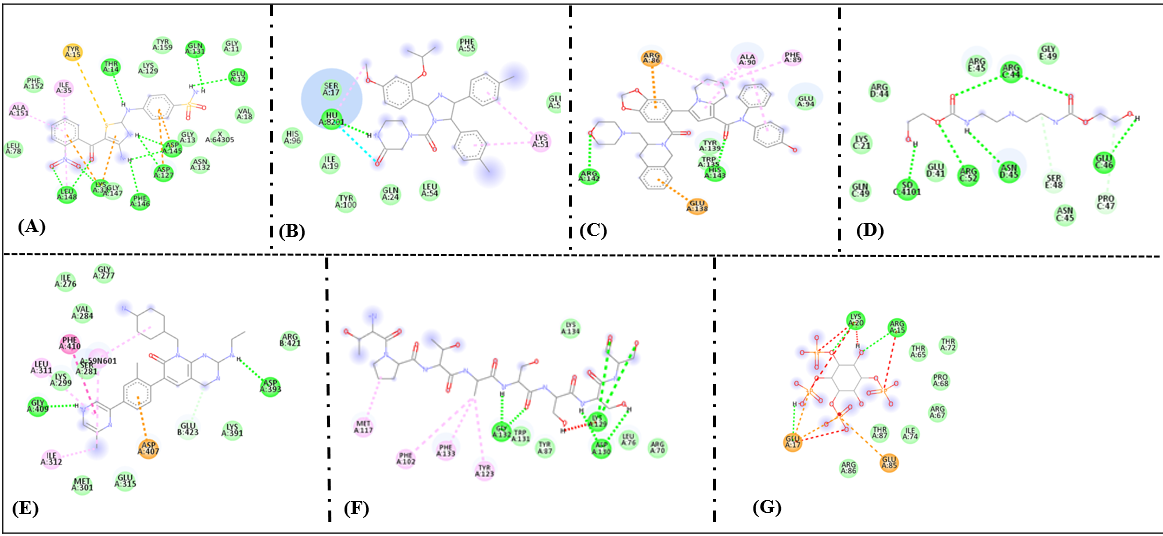


**Keys**: **(A)** CDK2-NL1; **(B)** MDM2-NL2; **(C),** BCL2-NL3; **(D),** Caspase-9-NL4; **(E),** P21- NL5 (PF3758309); **(F),** TP53-NL6 and **(G),** AKT1-NL7.

**Supplementary Figure S4.** A to G represent the 2D structure of redocking results for the native ligand-receptor interactions.

**Supplementary Table S8**. Binding affinities and receptor-ligand interactions of redocked ligands with selected hub target genes.

| Complex | Binding energy (kcal/mol) | Hydrogen bond | Hydrophobic interaction |
| --- | --- | --- | --- |
| CDK2-NL1 | -9.3 | Thr14A, Gln131A, Glu12A, Asp145A, Asp127A, Phe146A, Lys33A, Leu148A | Ala151A, Ile35A, Lys33A, Leu148A, Tyr15A, Asp127A, Asp145A |
| MDM2-NL2 | -7.3 | Hu8201A | Lys51A, Hu8201A |
| BCL2-NL3 | -8.1 | Arg142A, His143A | Arg86A, Ala90A, Phe89A, Glu138A |
| Caspase-9-NL4 | -9.3 | Arg44C, Arg52C, Asn45C, Glu46C, Ser48E, Pro47A |  |
| P21-NL5 | -8.5 | Gly409A, Glu423A, Asp393A | Leu311A, Phe410A, Ile312A, A59N601 |
| TP53-NL6 | -8 | Lys129A, Gly132A | Met117A, Phe102A, Phe133A, Tyr123A |
| AKT1-NL7 | -5.2 | Lys20A, Arg15A, Glu17A | Glu85A, Glu17A, Lys20A, Arg15A |

**Keys**: CDK2-NL1 (4-[[4-amino-5-(2-nitrobenzoyl)-1,3-thiazol-2 yl]amino] benzenesulfonamide); MDM2-NL2 (4-[(4S,5R)-4,5-bis(4-chlorophenyl)-2-(4-methoxy-2-propan-2-yloxyphenyl)-4,5-dihydroimidazole-1-carbonyl]piperazin-2-one); BCL2-NL3 ({N}-(4-hydroxyphenyl)-3-[6-[[(3~{S})-3-(morpholin-4-ylmethyl)-3,4-dihydro-1~{H}-isoquinolin-2-yl]carbonyl]-1,3-benzodioxol-5-yl]-~{N}-phenyl-5,6,7,8-tetrahydroindolizine-1-carboxamide); Caspase-9-NL4 (HEC); P21- NL5 (PF3758309); TP53-NL6 (3S)-N-(4'-carbamoyl[1,1'-biphenyl]-3-yl)-1-[4-(4-methylpiperazin-1-yl)pyridine-2-carbonyl]piperidine-3-carboxamide) and AKT1-NL7 (Inositol 1,3,4,5-tetrakisphosphate complexes
